# Death after hematopoietic stem cell transplantation: changes over calendar year time, infections and associated factors

**DOI:** 10.1038/s41409-019-0624-z

**Published:** 2019-08-27

**Authors:** Jan Styczyński, Gloria Tridello, Linda Koster, Simona Iacobelli, Anja van Biezen, Steffie van der Werf, Małgorzata Mikulska, Lidia Gil, Catherine Cordonnier, Per Ljungman, Diana Averbuch, Simone Cesaro, Rafael de la Camara, Helen Baldomero, Peter Bader, Grzegorz Basak, Chiara Bonini, Rafael Duarte, Carlo Dufour, Jurgen Kuball, Arjan Lankester, Silvia Montoto, Arnon Nagler, John A. Snowden, Nicolaus Kröger, Mohamad Mohty, Alois Gratwohl, for the Infectious Diseases Working Party EBMT

**Affiliations:** 1 Pediatric Hematology and Oncology, Collegium Medicum, Nicolaus Copernicus University Torun, Bydgoszcz, Poland; 2 Policlinico G.B. Rossi, Verona, Italy; 3 EBMT Data Office, Leiden, The Netherlands; 4 Università di Roma “Tor Vergata”, Roma, Italy; 5 Division of Infectious Diseases, University of Genoa (DISSAL) and Ospedale Policlinico San Martino, Genoa, Italy; 6 Medical University, Poznań, Poland; 7 Hôpital Henri Mondor, Assistance Publique-Hopitaux de Paris (AP-HP) and Paris-Est-Créteil University, Creteil, France; 8 Karolinska University Hospital, and Karolinska Institutet Stockholm, Stockholm, Sweden; 9 Hadassah University Hospital, Jerusalem, Israel; 10 Hospital de la Princesa, Madrid, Spain; 11 EBMT Activity Survey Office, Hematology, Department of Medicine, University Hospital, Basel, Switzerland; 12 Universitätsklinikum Frankfurt, Goethe-Universität, Frankfurt am Main, Germany; 13 Department of Hematology, Oncology and Internal Medicine, Medical University of Warsaw, Warsaw, Poland; 14 Università Vita-Salute San Raffaele, Milan, Italy; 15 Hospital Universitario Puerta de Hierro Majadahonda, Madrid, Spain; 16 Hematology Unit, G. Gaslini Children’s Institute, Genova, Italy; 17 Department of Haematology, University Medical Centre, Utrecht, The Netherlands; 18 Leiden University Hospital, Leiden, The Netherlands; 19 Department of Haemato-oncology, St Bartholomew’s Hospital, Barts Health NHS Trust, London, UK; 20 Chaim Sheba Medical Center, Tel-Hashomer, Israel; 21 Sheffield Teaching Hospitals NHS Foundation Trust, Sheffield, UK; 22 Department of Stem Cell Transplantation, University Hospital Eppendorf, Hamburg, Germany; 23 Department of Hematology, Hospital Saint Antoine, Paris, France; 24 Hematology, Medical Faculty, University of Basel, Basel, Switzerland

**Keywords:** Epidemiology, Risk factors

## Abstract

Information on incidence, and factors associated with mortality is a prerequisite to improve outcome after hematopoietic stem cell transplantation (HSCT). Therefore, 55′668 deaths in 114′491 patients with HSCT (83.7% allogeneic) for leukemia were investigated in a landmark analysis for causes of death at day 30 (very early), day 100 (early), at 1 year (intermediate) and at 5 years (late). Mortality from all causes decreased from cohort 1 (1980–2001) to cohort 2 (2002–2015) in all post-transplant phases after autologous HSCT. After allogeneic HSCT, mortality from infections, GVHD, and toxicity decreased up to 1 year, increased at 5 years; deaths from relapse increased in all post-transplant phases. Infections of unknown origin were the main cause of infectious deaths. Lethal bacterial and fungal infections decreased from cohort 1 to cohort 2, not unknown or mixed infections. Infectious deaths were associated with patient-, disease-, donor type, stem cell source, center, and country- related factors. Their impact varied over the post-transplant phases. Transplant centres have successfully managed to reduce death after HSCT in the early and intermediate post-transplant phases, and have identified risk factors. Late post-transplant care could be improved by focus on groups at risk and better identification of infections of “unknown origin”.

## Introduction

Besides the risk of relapse, hematopoietic stem cell transplantation (HSCT) remains associated with significant early and late treatment related mortality (TRM). Infections, toxicity, and (after allogeneic HSCT only), graft-vs.-host disease (GVHD) are the main causes of death. An earlier EBMT (European Society for Blood and Marrow Transplantation) analysis of patients with good risk leukemia transplanted between 1980–2001 had shown a significant increase in the 5-year survival rate from the 1980ies to the 1990ies, primarily due to a marked reduction of infectious deaths [1]. Since then, HSCT has become established as a valuable treatment option. The number of transplants has substantially increased [2–4], indications for HSCT have broadened [5], and new technologies have been introduced [4, 6–8]. It is estimated today that more than 1.4 million transplants have been performed so far worldwide; about 70′000 patients are now being treated annually with HSCT, half of them in Europe [3].

The improvement in outcome in the 1990ies has been confirmed by a single centre study for the years 1993–2007 in the United States. It showed a reduction of deaths from organ damage, infections, and severe acute GVHD [2]. No in depth large analysis has been conducted since. New antibiotics, new antifungal and antiviral agents have been introduced [9–14]. The large expansion of unrelated donor registries and the use of haploidentical transplants have given access to donors for many more patients [4]. The introduction of reduced-intensity conditioning transplants made HSCT accessible for elderly and frail patients [6]. Still, it remains unknown whether deaths from infections continued to decline since 2001. Furthermore, factors associated with death from infections, a prerequisite for further improvement, are largely unknown. We therefore designed the study to analyse the causes of deaths after HSCT over calendar year time, at specified post-transplant time phases, and searched for factors associated with it. We hypothesized that the key factors differ depending on the time phase after HSCT, both after allogeneic and autologous HSCT.

## Patients and methods

### Study design

This retrospective, observational study included all patients with HSCT from all donor types and stem cell sources for acute lymphoblastic leukemia (ALL), acute myeloid leukemia (AML) or chronic myeloid leukemia (CML) between 1980 and 2015, reported by 588 centers from 51 countries to the EBMT database. This patient selection and the two time periods (cohort 1 from 1980–2001and cohort 2 from 2002–2015) were based on a previous study, but did include now all disease stages, not only first CR patients [1]. There were no exclusion criteria. All data collection was performed by the IDWP Data Office (Leiden) according to EBMT guidelines (http://www.ebmt.org/retrospective-studies).

The analysis followed a stepwise approach. First, the data cohort was set up: all transplants in the EBMT data file from 1980 to 2015 were retrieved and assessed for completeness as specified below. In the second step, this file with complete information was closed as of January 1^st^ 2017, and recoded for the specific new grouping of donor type and centre and country specific economic parameters. Data were verified, and the descriptive analysis was performed.

All EBMT teams are required to obtain patients’ consent for data transfer to EBMT and to have internal review board approval for their transplant programs. The data set was locked and anonymized. No centers were contacted for missing information. No additional ethics approval was mandated.

### Study population

The study comprised a total number of 114′491 patients with HSCT, 95′789 (84%) allogeneic and 18′702 (16%) autologous, for AML (57.2%), ALL (25.4%), or CML (17.4%); of these 42′997 (37.6%) were in cohort 1, and 71′494(62.4%) in cohort 2. There were 56.5% male, 43.5% female patients with a median age of 37 years (0–84 years range) (Table 1).

**Table 1:** Patients population

	Year of last transplant				Total		*p* value
	1980–2001		2002–2015				
	*N*	%	*N*	%	*N*	%	
	42997	37.55	71494	62.45	114491	100.00	
Sex							
Male	24656	57.34	40038	56.00	64694	56.51	<0.0001
Female	18341	42.66	31456	44.00	49797	43.49	
Age at last HSCT (years)							
Median	33.1		41.0		37.4		<0.0001
Range	0.2–77.9		0.0–83.8		0.0–83.8		
Mean (SD)	32.24 (15.04)		38.95 (18.14)		36.43 (17.35)		
N obs	42972		71465		114437		
Underlying disease							
AML	20132	46.82	45386	63.48	65518	57.23	<0.0001
ALL	9431	21.93	19652	27.49	29083	25.40	
CML	13434	31.24	6456	9.03	19890	17.37	
Stage at last transplant							
early	27495	63.95	43946	61.47	71441	62.40	<0.0001
late	15502	36.05	27548	38.53	43050	37.60	
Type of HSCT							
Allogeneic	31128	72.40	64661	90.44	95789	83.67	<0.0001
Autologous	11869	27.60	6833	9.56	18702	16.33	
Stem cell source							
BM	30218	70.28	15466	21.63	45684	39.90	<0.0001
PB	12442	28.94	53829	75.29	66271	57.88	
CB	337	0.78	2199	3.08	2536	2.22	
T-cell depletion							
no	14097	62.24	30792	50.67	44889	53.81	<0.0001
yes in vivo, no ex vivo	2881	12.72	26488	43.59	29369	35.21	
yes ex vivo, no in vivo	3201	14.13	1357	2.23	4558	5.46	
yes in vivo + ex vivo	2472	10.91	2129	3.50	4601	5.52	
Conditioning regimen							
standard	32708	96.38	45487	70.77	78195	79.62	<0.0001
reduced	1230	3.62	18788	29.23	20018	20.38	
First HSCT							
No	2906	6.76	6342	8.87	9248	8.08	<0.0001
Yes	40091	93.24	65152	91.13	105243	91.92	
Geographic regions							
north-west	26319	61.21	38629	54.03	64948	56.73	<0.0001
south	14470	33.65	25987	36.35	40457	35.34	
east	2208	5.14	6878	9.62	9086	7.94	
GNI per capita 2015^a^							
High income	41424	96.34	63712	89.12	105136	91.83	<0.0001
Upper middle income	1565	3.64	7671	10.73	9236	8.07	
Lower middle income	8	0.02	111	0.16	119	0.10	
JACIE accreditation 2016							
accredited	22935	53.34	36420	50.94	59355	51.84	<0.0001
expired	7827	18.20	8439	11.80	16266	14.21	
withdrawn	279	0.65	369	0.52	648	0.57	
not accredited	11956	27.81	26266	36.74	38222	33.38	
Centre experience^b^							
0–4	6295	14.64	2236	3.13	8531	7.45	<0.0001
5–19	31026	72.16	29652	41.47	60678	53.00	
20+	5676	13.20	39606	55.40	45282	39.55	
Centre size^c^							
0–4	14940	34.75	14965	20.93	29905	26.12	<0.0001
5–19	24345	56.62	37794	52.86	62139	54.27	
20+	3712	8.63	18735	26.20	22447	19.61	

^a^Atlas methodology

^b^calculated from ProMISE: number of years performing transplants until the year of transplant of the patient

^c^calculated from ProMISE: number of transplants with the same disease and same year of transplant as the patient

All the differences resulted statistically significant (*p* < 0.0001)

### Working definitions

Death after transplant was categorized as previously defined [1, 15] into death from relapse (RM, relapse mortality: any death after relapse) and death from causes other than relapse (GVHD, toxicity, infection, other and unknown causes). Infectious deaths were analysed as total, and split by bacterial, fungal, viral, parasitic, mixed, and unknown infections.

Disease stage was defined as early disease (CR1 in ALL and AML; first CP in CML) or late disease (all other: ≥CR2, progression, refractory/relapsed).

Donor type was analysed in 10 types as previously described: autologous, syngeneic, HLA-identical sibling donor HY^-^, HLA-identical sibling donor HY^+^, matched family donor HY^-^, matched family donor HY^+^, matched unrelated donor HY^-^, matched unrelated donor HY^+^, mismatched unrelated donor, mismatched family donor (HY^+^ refers to a female donor for a male recipient; HY^-^ to all other donor-recipient sex combinations) [16]. Matching was used as classified by the reporting team, at the time of first report.

Acute GVHD was graded according to previously published criteria [17]. Chronic GVHD was graded as limited or extensive.

Centre specific microeconomic and country specific macroeconomic factors were integrated as previously described [18]. Centre size was defined for each transplant by the number of transplants performed in the centre for the main disease of the patient in the year of the transplant (0–4, 5–19, 20+ transplants). Centre experience in years was defined by the number of years that the centre had performed transplants until the year of the transplant of the patient (0–4, 5–19, 20+ years of program). Country macroeconomic status was defined by gross national income (GNI/capita) in 2016: high income, upper middle income, lower middle income (source: www.worldbank.org). Geographical regions were defined as: north-western eastern and southern (see Table 3A and Table 3B for details). JACIE accreditation status (accredited, expired, withdrawn and not accredited) in 2016 was used.

### Statistical analysis

The primary endpoint was to compute the cumulative incidence (CumInc) of overall post-transplant mortality in cohorts 1 and 2. The CumInc of death was reported at specified time phases after the transplant (very early: day +30, early: day +100, intermediate: +1 year and late: +5 years) in a landmark approach. Analysis at day +30 included all patients, the analyses at day +100, year +1, year +5 included patients alive at day +30, day +100, year +1, respectively.

Secondary endpoints were, as in the previous report [1], the CumInc of mortality due to relapse, GVHD, infection, other and unknown causes. GVHD was used as “any GVHD ever”, not separated by acute or chronic GVHD. The cumulative incidence estimator was applied, with the Gray test being used to compare different groups. Differences in CumInc rates without overlapping of the 95%CI’s were considered as significant.

The cause-specific Cox model was used to detect the factors related to the infection death for the four post-transplant phases. The death due to infection was considered as event of interest and death due to other causes as competing event.

Factors integrated into the analysis were sex and age of the patients, main diagnosis (ALL or AML or CML), stage of the disease, donor/recipient sex matching (HY^-^ vs HY^+^), type of donor (HLA-matched sibling vs other donor types), type of conditioning (RIC vs MAC), source of stem cells (PB vs BM vs CB), year and number of transplantation and presence or absence of in vivo and ex vivo T-cell depletion, and macro- and microeconomic center data as explained above. Factors with a *p*-value < 0.2 from the univariate analysis were included in the multivariate analysis. Factors with a *p* value of <0.05 were considered as significant.

Comparisons for categorical variables were done using the Fisher’s exact test or the *χ*^2^ test. The proportional hazard assumption was verified using graphical methods: scaled Schoenfeld [19] residuals and graphical checks proposed by Klein-Moeschberger [20] were performed without finding evidence of relevant violations. All the analyses were performed using the statistical software SAS (SAS Institute Inc., Cary, NC, USA) version 9.4.

## Results

### Patient population and demographic changes over calendar time

There were significant differences in absolute numbers, in patients, disease and donor characteristics, as well as in transplant procedure techniques between the two cohorts. Median age increased from 33.1 years to 41.0 years. The proportion and the absolute numbers of patients with AML and ALL increased, the ones of CML decreased. There were more allogeneic HSCT in the second cohort, more transplants with reduced intensity conditioning and more transplants with peripheral blood as stem cell source. Economic factors showed changes, with more transplants in the second cohort from eastern and lower income countries and within non accredited centres; there was an increase in transplants in more experienced centres by patient volume and years of transplants (Table 1).

### Main causes of death and changes over post-transplant phase and calendar time

**Fig. 1:**
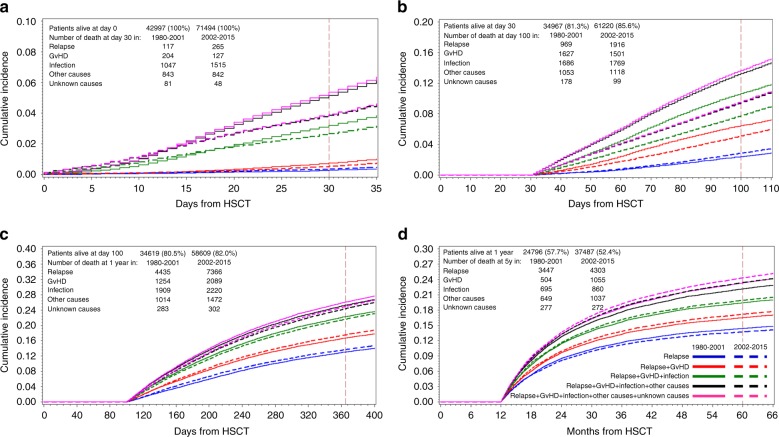
Cumulative incidences of mortality after HSCT over four post-transplant phases and from cohort 1 to cohort 2. The stacked curves for the four post-transplant phases for the two cohorts combined in landmark analysis are presented: **a** 30-day mortality; **b** 100-day mortality (for patients alive at day 30); **c** 1-year mortality (for patients alive at day 100); **d** 5-year mortality (for patients alive at 1 year)

**Fig. 2:**
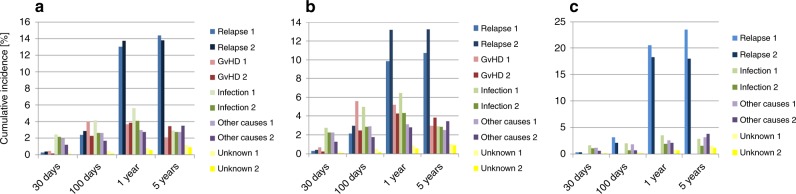
Main causes of death and of infectious deaths after HSCT. Main cause of death (in %)by post-transplant phase and by cohorts 1 and 2 (1 = cohort 1980–2001; 2 = cohort 2002–2015): **a** all patients; **b** allo-HSCT patients; **c** auto-HSCT patients. Cumulative incidences of mortality for the respective cause of death during the 4 post-transplant time periods, day 0 to day 30, day 30 to day 100, day 100 to 1 year, and 1 year to 5 years (see methods section for details), are shown

**Fig. 3:**
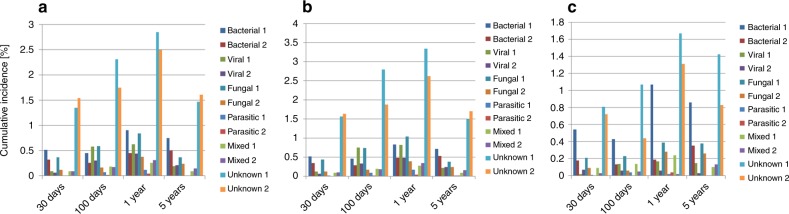
Main causes of death and of infectious deaths after HSCT. Causes of infectious deaths by post-transplant phase. Changes over time of causes of infectious deaths (in %) after HSCT for leukemia by post-transplant phase (1 = cohort 1980–2001; 2 = cohort 2002–2015): **a** all patients; **b** allo-HSCT patients; **c** auto-HSCT patients. Cumulative incidences of mortality for the respective cause of death during the 4 post-transplant time periods, day 0 to day 30, day 30 to day 100, day 100 to 1 year, and 1 year to 5 years (see methods section for details), are shown

A total of the 55′668 patients were reported as having died, 52′448 thereof during the observation period (45.8% of all patients; 43′930 after allogeneic, 8′518 after autologous HSCT): 22′518 (42.9%) died from relapse, 8′361 (15.9%) from GvHD, 11′701 (22.3%) from infections, 8028 (15.3%) from other, and 1540 (2.9%) from unknown causes (Table 2A and Table 2B). There were major differences between allogeneic (Table 2A, S5) and autologous (Table 2B, S6) HSCT, regarding main causes of death, changes over post-transplant phases and changes over calendar year time (Figs. 1–3, Fig. S1A). Main cause of death was relapse after both, allogeneic (38.7%) and autologous (64.5%), followed by infections (23.8% allogeneic; 14.8% autologous), and GvHD (19.0% allogeneic only). After allogeneic HSCT, 15′418 (35.1%) of all deaths occurred during the first 100 days, 10′174 (23.0%) between 1 and 5 years after the transplant, in contrast to autologous HSCT with 1587 (18.6%) of deaths within the first 100 days and 2925 (34.3%) of deaths between 1 and 5 year post-transplant.

**Table 2A:** Causes of deaths in 114′491 patients with leukemia and HSCT between 1980 and 2015, and cumulative incidences of deaths depending on patient cohort and time period after transplant. Patients after allo-HSCT

Time period	Cohort	Total at entry	Relapse		GvHD		Infection		Other causes		Unknown		Total	
			N D	Cum Inc + CI	N D	Cum Inc + CI	N D	Cum Inc + CI	N D	Cum Inc + CI	N D	Cum Inc + CI	N D	Cum Inc + CI
30 days	1	31128	81	0.26** (0.21–0.32)	204	0.66*** (0.57–0.75)	849	2.73*** (2.56–2.92)	703	2.26*** (2.10–2.43)	68	0.22*** (0.17–0.28)	1905	6.13*** (5.87–6.40)
	2	64661	242	0.38 (0.33–0.43)	127	0.20 (0.17–0.24)	1442	2.25 (2.13–2.36)	803	1.25 (1.17–1.34)	40	0.06 (0.05–0.08)	2654	4.14 (3.98–4.29)
100 days	1	29144	616	2.12*** (1.96–2.29)	1627	5.60*** (5.34–5.87)	1455	5.00*** (4.76–5.26)	844	2.90*** (2.71–3.10)	145	0.50*** (0.42–0.59)	4687	16.12*** (15.70–16.55)
	2	61164	1783	2.96 (2.83–3.10)	1501	2.49 (2.37–2.61)	1722	2.85 (2.72–2.98)	1074	1.78 (1.67–1.88)	92	0.15 (0.12–0.19)	6172	10.23 (9.99–10.47)
1 year	1	24275	2362	9.85*** (9.47–10.23)	1254	5.21*** (4.94–5.50)	1550	6.45*** (6.14–6.76)	747	3.11° (2.90–3.33)	204	0.85*** (0.74–0.97)	6117	25.47° (24.92–26.02)
	2	52756	6392	13.18 (12.88–13.48)	2089	4.28 (4.10–4.46)	2119	4.34 (4.17–4.53)	1358	2.79 (2.65–2.94)	263	0.54 (0.48–0.61)	12221	25.14 (24.75–25.52)
5 years	1	17630	1831	10.71*** (10.25–11.18)	504	2.95*** (2.71–3.22)	496	2.89^§^ (2.65–3.15)	432	2.54*** (2.32–2.79)	176	1.04° (0.89–1.20)	3439	20.14*** (19.54–20.74)
	2	33714	3714	13.26 (12.85–13.67)	1055	3.85 (3.62–4.08)	808	2.85 (2.66–3.05)	921	3.45 (3.23–3.68)	237	0.90 (0.79–1.03)	6735	24.31 (23.79–24.83)
Overall all study patients	1	31128	5278	16.29*** (15.87–16.71)	3763	11.75*** (11.39–12.11)	4475	14.20*** (13.82–14.60)	3380	8.93*** (8.61–9.25)	941	1.96*** (1.81–2.13)	17837	53.13*** (52.57–53.70)
	2	64661	12371	23.10 (22.73–23.47)	4898	8.74 (8.50–8.99)	6178	10.56 (10.30–10.81)	4416	7.58 (7.35–7.81)	723	1.26 (1.16–1.36)	28586	51.23 (50.79–51.68)

Time period: refers to the time period from day of transplant (see methods for details)

Cohort: refers to the two time periods 1980–2001 (cohort 1) or 2002–2015 (cohort 2)

Total at entry: refers to the number of patients at risk, at entry to the respective time period,

e.g., day 0 for 30 days period, day 30 for 100 days period, day 100 for 1 year period, and 1 year for 5 years period.

N D: number of deaths during the respective time period

Cum Inc + CI: Cumulative Incidence, and 95% confidence intervals. For full details see supplementary Tables 5 and 7.

Statistical significance in Cum Inc between cohort 1 and 2: § = n.s.; ° = < 0.05; * = < 0.01; ** = < 0.001; *** = < 0.0001 (i.e. risk of death from respective cause decreased/increased from cohort 1 to cohort 2)

**Table 2B:** Causes of deaths in 114′491 patients with leukemia and HSCT between 1980 and 2015, and cumulative incidences of deaths depending on patient cohort and time period after transplant. Patients after auto-HSCT

Time period	Cohort	Total at entry	Relapse		Infections		Other causes		Unknown		Total	
			N D	Cum Inc + CI	N D	Cum Inc + CI	N D	Cum Inc + CI	N D	Cum Inc + CI	N D	Cum Inc + CI
30 days	1	11869	36	0.31§ (0.22–0.42)	198	1.68** (1.46–1.93)	140	1.19*** (1.01–1.40)	13	0.11§ (0.06–0.19)	387	3.29*** (2.98–3.63)
	2	6833	23	0.34 (0.23–0.51)	73	1.09 (0.86–1.36)	39	0.58 (0.42–0.79)	8	0.12 (0.06–0.23)	143	2.14 (1.81–2.50)
100 days	1	11336	353	3.14*** (2.83–3.48)	231	2.05*** (1.80–2.32)	209	1.86*** (1.62–2.12)	33	0.29° (0.21–0.41)	826	7.34*** (6.87–7.83)
	2	6459	133	2.12 (1.79–2.50)	47	0.74 (0.55–0.98)	44	0.70 (0.51–0.93)	7	0.11 (0.05–0.22)	231	3.67 (3.23–4.16)
1 year	1	10344	2073	20.52** (19.74–21.32)	359	3.54*** (3.20–3.92)	267	2.63* (2.33–2.96)	79	0.78§ (0.62–0.97)	2778	27.48*** (26.61–28.35)
	2	5853	974	18.31 (17.28–19.37)	101	1.89 (1.55–2.28)	114	2.12 (1.76–2.54)	39	0.74 (0.53–1.00)	1228	23.06 (21.93–24.21)
5 years	1	7166	1616	23.51*** (22.51–24.52)	199	2.90*** (2.52–3.31)	217	3.18§ (2.79–3.62)	101	1.51§ (1.23–1.82)	2133	31.09*** (29.99–32.20)
	2	3773	589	18.04 (16.72–19.40)	52	1.59 (1.20–2.07)	116	3.80 (3.16–4.53)	35	1.14 (0.81–1.57)	792	24.57 (23.07–26.10)
Overall all study patients	1	11869	4349	37.01*** (36.10–37.92)	1011	8.72*** (8.21–9.26)	1018	7.41*** (6.94–7.91)	355	2.07§ (1.82–2.35)	6733	55.22*** (54.28–56.15)
	2	6833	1778	32.77 (31.47–34.08)	277	4.75 (4.21–5.33)	356	6.02 (5.38–6.71)	101	1.75 (1.41–2.15)	2512	45.29 (43.88–46.69)

Time period: refers to the time period from day of transplant (see methods for details)

Cohort: refers to the two time periods 1980–2001 (cohort 1) or 2002–2015 (cohort 2)

Total at entry: refers to the number of patients at risk, at entry to the respective time period,

e.g., day 0 for 30 days period, day 30 for 100 days period, day 100 for 1 year period, and 1 year for 5 years period.

N D: number of deaths during the respective time period

Cum Inc + CI: Cumulative Incidence, and 95% confidence intervals. For full details see supplementary Tables 5 and 7.

Statistical significance in Cum Inc between cohort 1 and 2: § = n.s.; ° = < 0.05; * = < 0.01; ** = < 0.001; *** = < 0.0001 (i.e. risk of death from respective cause decreased/increased from cohort 1 to cohort 2)

The changes in mortality from cohort 1 to cohort 2 differed between allogeneic and autologous HSCT for the four post-transplant periods. In the landmark analysis, the cumulative incidence of overall mortality decreased from cohort 1 to cohort 2 at +30d (*p* < 0.001), at +100d (*p* < 0.001), and at +1y (*p* < 0.001), but increased at +5y (*p* < 0.01) (Fig. 1, Table 2A and Table 2B, Table S1A). After autologous HSCT, the cumulative incidence for overall mortality decreased in each of the four post-transplant phases for each cause of death, including relapse except for “other” causes of death in very late phase (Table 2A and Table 2B, S1B). After allogeneic HSCT, mortality from GVHD, and other causes decreased in the very early, early, and intermediate phases, but increased in the late phase for GVHD and for other causes (Fig. 2, Table 2A and Table 2B, S1C). Mortality from relapse increased in all post-transplant phases. As a result, overall mortality decreased in the very early (+d30; *p* < 0.0001) and early phase (+d100; *p* < 0.0001), but increased in the late phase (+5y: *p* < 0.0001) (Table 2A and Table 2B, S1C).

### Causes of infectious death and changes over post-transplant phase and calendar year time

A total of 11′941 patients died of infectious complications. The majority of lethal infections (59.4%) were of unknown etiology, 14.7% were of bacterial, 10.6% of fungal, 9.2% of viral, 1% of parasitic, and 5.2% of mixed origin (Fig. 3, Fig. S1B). Mortality from mixed/unknown infections increased from cohort 1 to cohort 2 in the very early phase (+30d: 1.35; 95%CI 1.25–1.47 vs 1.54; 95%CI 1.45–1.63). Mortality from bacterial, viral, fungal and parasitic infections decreased in very early, early, and intermediate phases (Fig. 3, Fig. S1B). In the late phase, mortality from bacterial and fungal infections decreased, while mortality from viral, mixed or unknown infectious etiology did not change (Table S1A). The pattern of infectious deaths, including bacterial, viral, fungal and parasitic infections was similar for allo- and auto-HSCT, but with a distinct and constantly lower CumInc for all types of infections at all phases after HSCT for auto-HSCT (Tables S1BC, Tables S2-S6).

### Factors associated with death from infections

Factors associated with death from infection after HSCT related to patient, disease, donor-type, stem cell source, year of transplant and center specific macro- or micro-economic properties. Their impact varied significantly depending on main donor type (autologous vs. allogeneic) and the phase after transplant (Table 3A and Table 3B, Fig. S2AB). Increasing age of the patient, advanced disease stage, donor type, and second or later transplant were associated with increased risk of infectious death in all phases and for all donor types. Patient sex showed no associations at all.

**Table 3A:**
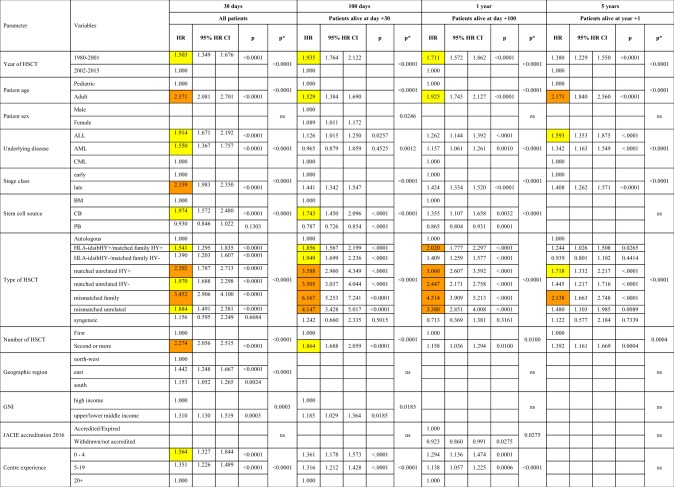
Factors associated with death from infection after HSCT in multivariate analysis. Factors associated with death from infection after HSCT in all patients

Centre size was defined for each transplant by the number of transplants performed in the center for the main disease of the patient in the year of the transplant (0–4, 5–19, 20 + transplants). Centre experience in years was defined by the number of years that the center had performed transplants until the year of the transplant of the patient (0–4, 5–19, 20 + years of program). Country macroeconomic status was defined by gross national income (GNI/capita) in 2016 (high income, upper middle income, lower middle income) (source: www.worldbank.org). Geographical regions were defined as: north-western (Austria, Belgium, Denmark, Finland, France, Germany, Luxembourg, Netherlands, Norway, Sweden, Switzerland, UK), eastern (Bulgaria, Croatia, Czech Republic, Hungary, Lithuania, Macedonia, Poland, Romania, Russia, Slovakia) and southern (Cyprus, Greece, Israel, Italy, Portugal, Spain, Turkey, and other EBMT countries). JACIE accreditation status (accredited, expired, withdrawn and not accredited) in 2016 was used. HR > 2.0 is highlighted in orange; HR > 1.5 is highlighted in yellow.

**Table 3B:**
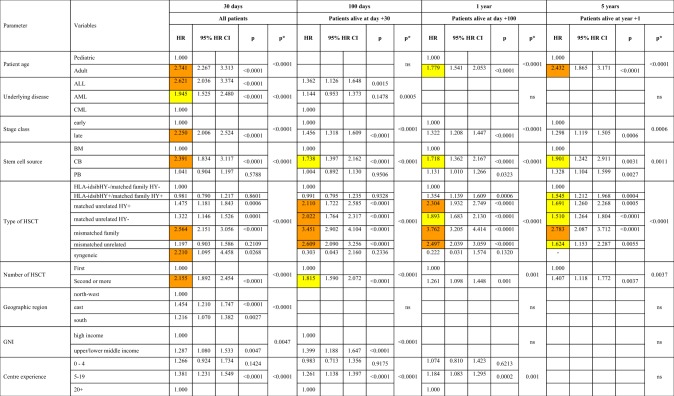
Factors associated with death from infection after allogeneic HSCT only in the second cohort

Peripheral blood as stem cell source was associated with less infectious deaths in all post-transplant phases after autologous HSCT; it was associated with more infectious deaths after allogeneic HSCT in the late phase, reflecting the higher probability of chronic GvHD with peripheral blood. This same association with GvHD in the late phase is reflected by the higher rate of deaths from infections in the HY^+^ donor recipient combinations (Table 3A and Table 3B, Fig. S2AB).

Of note, country specific macro-economic factors, GNI/capita and geographic region, were associated only during the very early and early post-transplant phase; the same applied to centre specific micro-economic factors. Centres with more than 20 years of disease specific transplant experience had significantly less infectious death in the early and intermediate post-transplant phases. No significant association could be documented between death from infections and JACIE accreditation status, in contrast to the association with overall mortality.

## Discussion

The results of this comprehensive study are clear: the European transplant teams have successfully managed to reduce all-cause mortality after autologous HSCT at all post-transplant time phases. In allogeneic HSCT, they were successful in reducing deaths from GVHD, infections and other causes in the very early and early post-transplant time phases, despite an increase in the patient pre-transplant risk profile. In contrast, data did not show a reduction of death from relapse after allogeneic HSCT, and no reduced mortality in the late post-transplant phase. The latter observation is of concern, but indicates areas for improvements.

The analysis confirmed well established disease, patient, donor, transplant and center-related risk factors for death from all causes and from infection after transplant [1, 2, 21]. Novel is the observation that not all factors are equally relevant during all post-transplant time phases. Advanced disease stage at time of transplant remains associated with increased risk of mortality from all causes throughout the whole post-transplant phase, and so is increasing age of the recipient, with the exception of the very early phase where other factors dominate. During all post-transplant phases, allogenicity dominates. Of note, the HY^+^ effect adds bearing on mortality primarily after day 100; the early beneficial effect of peripheral blood stem cells reverts to a detrimental effect beyond 1 year. As observed earlier, accreditation status of the center is associated with mortality of the patients and with overall improvement over calendar year time [22].

The same risk factors were associated with death from infections. Late disease stage was in all post-transplant phases associated with more infectious deaths. Increasing age contributed to risk of death from infection in a hierarchical effect by decade. Of interest, despite an increase in age in cohort 2, deaths from infections were still reduced, reflecting the possible benefit of better management of infectious complications, through novel diagnostic methods, drugs and guidelines [9–11, 23–25]. Cord blood as a stem cell source was associated with a higher rate of infectious deaths in the first three post-transplant phases, but no longer after 1 year. Peripheral blood as a stem cell source was associated with a lower rate of deaths in the first three phases, not in the last. These results might have been influenced by an additional, indirect late GVHD effect of peripheral blood stem cells. This is supported by the increased risk of infectious deaths in HY^+^ donor recipient combinations. Of note, disease specific center experience in years was strongly associated with reduced infectious deaths in the early post-transplant phase [22].

There are major caveats in this study. It looks at very heterogeneous data over a long time period with many changes in disease indications, choice of donor type, stem cell source. over calendar year time. Data were derived from a multitude of centers from many countries with different micro- and macroeconomic backgrounds. Some data, such as cytogenetic profiles were not in the data set. Some decisions had to be arbitrary, such as the use of economic factors as of the year 2016. Centers had varying attitudes regarding data collection, and potentially different interpretations of “cause of death”. Simply, we used the data as they were reported to the database, and we accepted the information as given by the center, including the high rate of deaths and infectious deaths of “unknown” origin. Still, the consistency of the findings in the four post-transplant phases, and the confirmation of key risk factor elements are strong arguments that the data are valid. They were as well in line with our pre-set hypotheses, that factors associated with overall mortality and from infections would differ depending on the post-transplant phase. This was the case, but is of concern. Mortality was reduced early post-transplant, but increased in the late phase after allogeneic HSCT. Hence, improvements were more rapidly visible than deteriorations; the increase in late mortality years after the transplant might not be recognized to the same extent, with patients frequently at distance from the center. Lethal infections caused by bacteria and fungi were reduced at any time point, but not infections of unknown origin.

This high rate of “unknown “ infections is intriguing and of prime concern. The constant pattern in all reporting centers, over calendar year time and over all post-transplant phases indicates that it might be a real entity, but without the same awareness as any specified infection. There is no information in the data set, whether the appropriate tests were not done, were not successful, or whether organ-related infections, such as pneumonia with negative microbiological work up were simply classified as such [15]. It will be essential for the future to learn whether “unknown” includes hitherto unknown infectious agents, or might be a set of multiple immune response syndromes. The focus on specified risk groups will support such strategies. Late infectious mortality as an entity remains of concern. It presents a complicated issue, probably resulting from the negative effects of many variables such as GVHD, inadequate immune recovery, poor graft function, increasing co-morbidities with increasing age. It might reflect as well the variable expertise of health care providers in the different transplant centers for the long-term follow-up of their patients.

What are the consequences of this report? The positive message is that outcomes were and can be improved. With similar efforts in late post-transplant care, it will be possible to improve outcome beyond 1 year as well. The first step is to become aware of the reality, to shift efforts, and to focus on preventable and reversible late complications. Strategies will need to include efforts for better identification of causes and treatment of mixed and “unknown” infections through collaborative efforts. Older patients, those with a second or later transplant and those with mismatched allogeneic transplants remain at higher risk, even years after transplant. They might require targeted follow-up programs. Prospective studies assessing causes of death 5 years or more after HSCT could help to characterize and possibly to target efforts to prevent late mortality. Finally, the major role of “allogeneicity” in death after allogeneic compared to autologous HSCT might lead to reconsider more carefully risk adapted indications for allogeneic HSCT.

Public health bodies and transplant teams need to recognize that fatal events can occur many months or years after the transplant procedure. Early detection and rapid treatment, particularly of infections, requires allocation of resources. Support includes data collection and data analysis which are integral parts of any transplant therapy [26]. Recent FACT-JACIE standards have incorporated long-term follow-up strategies between specialist transplant centers and local health-care services. Given the long time horizons, this will require support in planning and resources by public health bodies [27–29]. Furthermore, in the era of precision medicine and targeted therapies, HSCT has to provide best outcome regarding overall survival, quality of life and costs compared to any other treatment strategy [16]. Careful and continuing analysis of large data sets will help to achieve this goal.

## Supplementary information

Supplementary material
