# Supplementary material for: Death after hematopoietic stem cell transplantation: changes over calendar year time, infections and associated factors
Source: Bone Marrow Transplant. 2019 Aug 27;55(1):126–36. doi: 10.1038/s41409-019-0624-z (PMC6957465; doi:10.1038/s41409-019-0624-z)
Supplement: Supplementary file 1 — Supplementary material [file 41409_2019_624_MOESM1_ESM.docx]

**Death after hematopoietic stem cell transplantation: changes over calendar year time, infections and associated factors**

Jan Styczynski^1^, Gloria Tridello^2^, Linda Koster^3^, Simona Iacobelli^4^, Anja van Biezen^3^, Steffie van der Werf^3^, Malgorzata Mikulska^5^, Lidia Gil^6^, Catherine Cordonnier^7^, Per Ljungman^8^, Diana Averbuch^9^, Simone Cesaro^2^, Rafael de la Camara^10^, Helen Baldomero^11^, Peter Bader^12^, Grzegorz Basak^13^, Chiara Bonini^14^, Rafael Duarte^15^, Carlo Dufour^16^, Jurgen Kuball^17^, Arjan Lankester^18^, Silvia Montoto^19^, Arnon Nagler^20^, John A Snowden^21^, Nicolaus Kröger^22^, Mohamad Mohty^23^, and Alois Gratwohl^24^ for the Infectious Diseases Working Party EBMT

**SUPPLEMENTARY METHODS**

**Study Design**

The analysis followed a stepwise approach. In the first step, the data cohort was set up: all transplants in the EBMT data file from 1980 to 2015 were retrieved and assessed for completeness of the information as specified below. In the second step, the separate data file with complete information was closed as of January 1st 2017, and recoded for the specific new grouping of donor type and centre and country specific economic parameters. Data were verified. In the third step, the descriptive analysis was performed.

All EBMT teams are required to obtain patients’ consent for data transfer to EBMT and to have internal review board approval for their transplant programs. The data set was locked at time of study start and anonymized. No centers were contacted for missing information. No additional ethics approval was mandated.

**SUPPLEMENTARY FIGURES**

**Figure-S1. Causes of death by main donor type and post-transplant phase.**

**Figure-S1A. Main causes of death**

**Figure-S1B. Causes of infectious deaths**

**Figure-S2. Factors associated with death from infection at four time points (30 days; 100 days; 1 year; 5 years):**

**Figure-S2A. All patients, both cohorts.**

Legend: MF, matched family; MU, matched unrelated; MMF, mismatched family; MMU, mismatched unrelated

**Figure-S2B. Allogeneic HSCT patients, cohort 2 only (2002-2015).**

Only factors with p<0.2 in the univariate analysis were included in the multivariate analysis; hence not all factors are depicted at all post-transplant phases. HRs obtained from the multivariate models with their correspondent confidence interval were reported. Non-significant variables were artificially set as HR=1. Legend: MF, matched family; MU, matched unrelated; MMF, mismatched family; MMU, mismatched unrelated, HR, hazard ratio.

**SUPPLEMENTARY TABLES**

**Table-S1A. Causes of death in entire cohort of patient**

**All patients**

|  | Year of last transplant | | | | | | | | Total | | | |
| --- | --- | --- | --- | --- | --- | --- | --- | --- | --- | --- | --- | --- |
|  | 1980-2001 | | | | 2002-2015 | | | |  |  |  |  |
|  | N=42997 | | | | N=71494 | | | | N=114491 | | | |
|  | 30 days | 100 days | 1 year | 5 years | 30 days | 100 days | 1 year | 5 years | 30 days | 100 days | 1 year | 5 years |
| **Main cause of death** |  | alive at day 30 | alive at day 100 | alive at 1 year |  | alive at day 30 | alive at day 100 | alive at 1 year |  | alive at day 30 | alive at day 100 | alive at 1 year |
| Relapse | 0.27  (0.23-0.33) | 2.41  (2.26-2.56) | 13.01 (12.66-13.37) | 14.37 (13.93-14.82) | 0.37 (0.33-0.42) | 2.88 (2.76-3.01) | 13.69 (13.40-13.98) | 13.76 (13.37-14.15) | 0.34 (0.30-0.37) | 2.70 (2.61-2.80) | 13.43 (13.21-13.66) | 14.03 (13.74-14.32) |
| GvHD | 0.48 (0.42-0.55) | 4.03 (3.85-4.23) | 3.66 (3.47-3.87) | 2.11 (1.93-2.30) | 2.14 (2.03-2.25) | 2.65 (2.53-2.77) | 4.10 (3.94-4.27) | 2.72 (2.54-2.91) | 0.29 (0.26-0.32) | 2.92 (2.82-3.03) | 3.78 (3.65-3.90) | 2.85 (2.71-2.99) |
| Infection | 2.44 (2.30-2.59) | 4.18 (3.99-4.38) | 5.59 (5.35-5.83) | 2.89 (2.69-3.11) | 2.14 (2.03-2.25) | 2.65 (2.53-2.77) | 4.10 (3.94-4.27) | 2.72 (2.54-2.91) | 2.25 (2.17-2.34) | 3.23 (3.12-3.33) | 4.67 (4.53-4.81) | 2.80 (2.66-2.94) |
| Other causes | 1.97 (1.84-2.10) | 2.61 (2.46-2.77) | 2.97 (2.79-3.15) | 2.73 (2.53-2.94) | 1.19 (1.11-1.27) | 1.67 (1.58-1.77) | 2.73 (2.59-2.87) | 3.49 (3.28-3.71) | 1.48 (1.41-1.55) | 2.03 (1.94-2.11) | 2.82 (2.71-2.93) | 3.14 (3.00-3.29) |
| Unknown | 0.19 (0.15-0.23) | 0.44 (0.38-0.51) | 0.83 (0.74-0.93) | 1.17 (1.04-1.31) | 0.07 (0.05-0.09) | 0.15 (0.12-0.18) | 0.56 (0.50-0.63) | 0.93 (0.82-1.05) | 0.11 (0.10-0.13) | 0.26 (0.23-0.29) | 0.66 (0.61-0.72) | 1.04 (0.95-1.13) |
| Total mortality | 5.35 (5.14-5.57) | 13.67 (13.34-14.01) | 26.06 (25.59-26.52) | 23.27 (22.73-23.81) | 3.95 (3.81-4.09) | 9.61 (9.39-9.83) | 24.93 (24.56-25.30) | 24.34 (23.85-24.83) | 4.48 (4.36-4.60) | 11.14 (10.95-11.33) | 25.36 (25.08-25.65) | 23.86 (23.49-24.22) |
|  |  |  |  |  |  |  |  |  |  |  |  |  |
| Bacterial | 0.52 (0.46-0.60) | 0.45 (0.39-0.52) | 0.90 (0.80-1.00) | 0.75 (0.65-0.87) | 0.32 (0.29-0.37) | 0.26 (0.23-0.31) | 0.45 (0.39-0.51) | 0.51 (0.44-0.60) | 0.40 (0.36-0.44) | 0.34 (0.30-0.37) | 0.62 (0.57-0.68) | 0.62 (0.55-0.68) |
| Viral | 0.09 (0.07-0.12) | 0.58 (0.51-0.66) | 0.63 (0.55-0.71) | 0.19 (0.14-0.26) | 0.06 (0.04-0.08) | 0.30 (0.26-0.35) | 0.44 (0.39-0.50) | 0.21 (0.16-0.26) | 0.07 (0.06-0.09) | 0.41 (0.37-0.45) | 0.51 (0.47-0.56) | 0.20 (0.17-0.24) |
| Fungal | 0.37 (0.32-0.43) | 0.59 (0.52-0.67) | 0.84 (0.75-0.94) | 0.37 (0.30-0.46) | 0.12 (0.09-0.15) | 0.16 (0.13-0.20) | 0.38 (0.33-0.43) | 0.24 (0.19-0.30) | 0.21 (0.19-0.24) | 0.32 (0.29-0.36) | 0.56 (0.51-0.61) | 0.30 (0.26-0.35) |
| Parasitic | 0.01 (0.00-0.03) | 0.07 (0.05-0.11) | 0.12 (0.09-0.16) | 0.01 (0.00-0.04) | 0.00 (0.00-0.01) | 0.02 (0.01-0.03) | 0.04 (0.02-0.06) | 0.01 (0.00-0.03) | 0.01 (0.00-0.01) | 0.04 (0.03-0.05) | 0.07 (0.05-0.09) | 0.01 (0.00-0.02) |
| Mixed | 0.09 (0.07-0.13) | 0.18 (0.14-0.22) | 0.26 (0.21-0.32) | 0.09 (0.06-0.13) | 0.09 (0.07-0.12) | 0.17 (0.14-0.20) | 0.31 (0.26-0.36) | 0.15 (0.11-0.20) | 0.09 (0.08-0.11) | 0.17 (0.15-0.20) | 0.29 (0.25-0.33) | 0.12 (0.10-0.15) |
| Unknown infection | 1.35 (1.25-1.47) | 2.31 (2.16-2.46) | 2.84 (2.67-3.02) | 1.47 (1.33-1.63) | 1.54 (1.45-1.63) | 1.74 (1.64-1.84) | 2.49 (2.36-2.63) | 1.61 (1.47-1.75) | 1.47 (1.40-1.54) | 1.95 (1.87-2.04) | 2.63 (2.52-2.73) | 1.55 (1.45-1.65) |

Provided values represent cumulative incidence (95%CI).

**Table-S1B. Causes of death in auto-HSCT patients**

**Autologous**

|  | Year of last transplant | | | | | | | | Total | | | |
| --- | --- | --- | --- | --- | --- | --- | --- | --- | --- | --- | --- | --- |
|  | 1980-2001 | | | | 2002-2015 | | | |  |  |  |  |
|  | N=11869 | | | | N=6833 | | | | N=18702 | | | |
|  | 30 days | 100 days | 1 year | 5 years | 30 days | 100 days | 1 year | 5 years | 30 days | 100 days | 1 year | 5 years |
| **Main cause of death** |  | alive at day 30 | alive at day 100 | alive at 1 year |  | alive at day 30 | alive at day 100 | alive at 1 year |  | alive at day 30 | alive at day 100 | alive at 1 year |
| Relapse | 0.31 (0.22-0.42) | 3.14 (2.83-3.48) | 20.52 (19.74-21.32) | 23.51 (22.51-24.52) | 0.34 (0.23-0.51) | 2.12 (1.79-2.50) | 18.31 (17.28-19.37) | 18.04 (16.72-19.40) | 0.32 (0.25-0.41) | 2.78 (2.54-3.03) | 19.77 (19.15-20.41) | 21.77 (20.97-22.59) |
| Infection | 1.68 (1.46-1.93) | 2.05 (1.80-2.32) | 3.54 (3.20-3.92) | 2.90 (2.52-3.31) | 1.09 (0.86-1.36) | 0.74 (0.55-0.98) | 1.89 (1.55-2.28) | 1.59 (1.20-2.07) | 1.47 (1.30-1.65) | 1.58 (1.40-1.77) | 2.97 (2.71-3.25) | 2.48 (2.19-2.80) |
| Other causes | 1.19 (1.01-1.40) | 1.86 (1.62-2.12) | 2.63 (2.33-2.96) | 3.18 (2.79-3.62) | 0.58 (0.42-0.79) | 0.70 (0.51-0.93) | 2.12 (1.76-2.54) | 3.80 (3.16-4.53) | 0.97 (0.84-1.12) | 1.44 (1.27-1.62) | 2.46 (2.22-2.71) | 3.36 (3.02-3.73) |
| Unknown | 0.11 (0.06-0.19) | 0.29 (0.21-0.41) | 0.78 (0.62-0.97) | 1.51 (1.23-1.82) | 0.12 (0.06-0.23) | 0.11 (0.05-0.22) | 0.74 (0.53-1.00) | 1.14 (0.81-1.57) | 0.11 (0.07-0.17) | 0.23 (0.17-0.31) | 0.76 (0.64-0.91) | 1.40 (1.18-1.64) |
| Total mortality | 3.29 (2.98-3.63) | 7.34 (6.87-7.83) | 27.48 (26.61-28.35) | 31.09 (29.99-32.20) | 2.14 (1.81-2.50) | 3.67 (3.23-4.16) | 23.06 (21.93-24.21) | 24.57 (23.07-26.10) | 2.87 (2.64-3.12) | 6.02 (5.68-6.38) | 25.96 (25.27-26.66) | 29.01 (28.12-29.91) |
|  |  |  |  |  |  |  |  |  |  |  |  |  |
| Bacterial | 0.54 (0.42-0.69) | 0.43 (0.32-0.56) | 1.07 (0.88-1.28) | 0.86 (0.66-1.10) | 0.18 (0.10-0.31) | 0.13 (0.06-0.25) | 0.19 (0.10-0.34) | 0.35 (0.18-0.61) | 0.41 (0.33-0.51) | 0.32 (0.24-0.41) | 0.76 (0.64-0.91) | 0.69 (0.55-0.87) |
| Viral | 0.02 (0.00-0.06) | 0.14 (0.09-0.23) | 0.17 (0.10-0.26) | 0.15 (0.08-0.26) | 0.07 (0.03-0.17) | 0.06 (0.02-0.16) | 0.06 (0.02-0.16) | 0.03 (0.00-0.02) | 0.04 (0.02-0.08) | 0.11 (0.07-0.17) | 0.13 (0.08-0.20) | 0.11 (0.06-0.19) |
| Fungal | 0.21 (0.14-0.31) | 0.23 (0.16-0.34) | 0.39 (0.28-0.52) | 0.38 (0.25-0.54) | 0.09 (0.04-0.19) | 0.06 (0.02-0.16) | 0.28 (0.17-0.46) | 0.26 (0.13-0.49) | 0.17 (0.12-0.24) | 0.17 (0.12-0.24) | 0.35 (0.27-0.45) | 0.34 (0.24-0.47) |
| Parasitic | 0.01 (0.00-0.05) | 0.04 (0.01-0.09) | 0.02 (0.00-0.07) | 0.00 | 0.00 | 0.00 | 0.04 (0.01-0.13) | 0.00 | 0.01 (0.00-0.03) | 0.02 (0.01-0.06) | 0.03 (0.01-0.06) | 0.00 |
| Mixed | 0.09 (0.05-0.17) | 0.14 (0.09-0.23) | 0.24 (0.16-0.35) | 0.10 (0.05-0.20) | 0.03 (0.01-0.11) | 0.05 (0.01-0.14) | 0.02 (0.00-0.11) | 0.13 (0.04-0.32) | 0.07 (0.04-0.12) | 0.11 (0.07-0.17) | 0.16 (0.11-0.23) | 0.11 (0.06-0.19) |
| Unknown infection | 0.81 (0.66-0.98) | 1.07 (0.90-1.28) | 1.67 (1.43-1.93) | 1.42 (1.16-1.72) | 0.72 (0.54-0.94) | 0.44 (0.30-0.63) | 1.31 (1.03-1.64) | 0.83 (0.56-1.19) | 0.78 (0.66-0.91) | 0.85 (0.72-0.99) | 1.55 (1.36-1.75) | 1.23 (1.03-1.46) |

Provided values represent cumulative incidence (95%CI).

**Table-S1C. Causes of death in allo-HSCT patients**

**Allogeneic**

|  | Year of last transplant | | | | | | | | Total | | | |
| --- | --- | --- | --- | --- | --- | --- | --- | --- | --- | --- | --- | --- |
|  | 1980-2001 | | | | 2002-2015 | | | |  |  |  |  |
|  | N=31128 | | | | N=64661 | | | | N=95789 | | | |
|  | 30 days | 100 days | 1 year | 5 years | 30 days | 100 days | 1 year | 5 years | 30 days | 100 days | 1 year | 5 years |
| **Main cause of death** |  | alive at day 30 | alive at day 100 | alive at 1 year |  | alive at day 30 | alive at day 100 | alive at 1 year |  | alive at day 30 | alive at day 100 | alive at 1 year |
| Relapse | 0.26 (0.21-0.32) | 2.12 (1.96-2.29) | 9.85 (9.47-10.23) | 10.71 (10.25-11.18) | 0.38 (0.33-0.43) | 2.96 (2.83-3.10) | 13.18 (12.88-13.48) | 13.26 (12.85-13.67) | 0.34 (0.30-0.38) | 2.69 (2.58-2.80) | 12.08 (11.84-12.32) | 12.29 (11.98-12.60) |
| GvHD | 0.66 (0.57-0.75) | 5.60 (5.34-5.87) | 5.21 (4.94-5.50) | 2.95 (2.71-3.22) | 0.20 (0.17-0.24) | 2.49 (2.37-2.61) | 4.28 (4.10-4.46) | 3.85 (3.62-4.08) | 0.35 (0.31-0.39) | 3.50 (3.38-3.62) | 4.58 (4.43-4.74) | 3.50 (3.33-3.67) |
| Infection | 2.73 (2.56-2.92) | 5.00 (4.76-5.26) | 6.45 (6.14-6.76) | 2.89 (2.65-3.15) | 2.25 (2.13-2.36) | 2.85 (2.72-2.98) | 4.34 (4.17-4.53) | 2.85 (2.66-3.05) | 2.41 (2.31-2.50) | 3.55 (3.43-3.67) | 5.03 (4.88-5.20) | 2.86 (2.71-3.02) |
| Other causes | 2.26 (2.10-2.43) | 2.90 (2.71-3.10) | 3.11 (2.90-3.33) | 2.54 (2.32-2.79) | 1.25 (1.17-1.34) | 1.78 (1.67-1.88) | 2.79 (2.65-2.94) | 3.45 (3.23-3.68) | 1.58 (1.50-1.66) | 2.14 (2.05-2.24) | 2.90 (2.77-3.02) | 3.09 (2.93-3.26) |
| Unknown | 0.22 (0.17-0.28) | 0.50 (0.42-0.59) | 0.85 (0.74-0.97) | 1.04 (0.89-1.20) | 0.06 (0.05-0.08) | 0.15 (0.12-0.19) | 0.54 (0.48-0.61) | 0.90 (0.79-1.03) | 0.11 (0.09-0.14) | 0.27 (0.23-0.30) | 0.64 (0.59-0.70) | 0.95 (0.86-1.05) |
| Total mortality | 6.13 (5.87-6.40) | 16.12 (15.70-16.55) | 25.47 (24.92-26.02) | 20.14 (19.54-20.74) | 4.14 (3.98-4.29) | 10.23 (9.99-10.47) | 25.14 (24.75-25.52) | 24.31 (23.79-24.83) | 4.79 (4.65-4.92) | 12.14 (11.93-12.36) | 25.23 (24.92-25.55) | 22.69 (22.30-23.08) |
|  |  |  |  |  |  |  |  |  |  |  |  |  |
| Bacterial | 0.52 (0.44-0.60) | 0.46 (0.39-0.55) | 0.83 (0.72-0.95) | 0.71 (0.60-0.85) | 0.34 (0.30-0.39) | 0.28 (0.24-0.32) | 0.48 (0.42-0.54) | 0.53 (0.45-0.62) | 0.40 (0.36-0.44) | 0.34 (0.30-0.38) | 0.59 (0.54-0.65) | 0.60 (0.53-0.67) |
| Viral | 0.12 (0.09-0.16) | 0.75 (0.65-0.85) | 0.82 (0.71-0.94) | 0.21 (0.15-0.29) | 0.06 (0.04-0.08) | 0.33 (0.28-0.38) | 0.48 (0.43-0.55) | 0.23 (0.18-0.29) | 0.08 (0.06-0.10) | 0.46 (0.42-0.51) | 0.59 (0.54-0.65) | 0.22 (0.18-0.27) |
| Fungal | 0.43 (0.36-0.51) | 0.73 (0.63-0.83) | 1.04 (0.91-1.17) | 0.37 (0.29-0.47) | 0.12 (0.10-0.15) | 0.17 (0.14-0.21) | 0.39 (0.34-0.45) | 0.24 (0.19-0.30) | 0.22 (0.19-0.25) | 0.35 (0.32-0.39) | 0.60 (0.55-0.66) | 0.29 (0.24-0.34) |
| Parasitic | 0.01 (0.00-0.03) | 0.09 (0.06-0.13) | 0.16 (0.11-0.22) | 0.02 (0.01-0.05) | 0.00 (0.00-0.01) | 0.02 (0.01-0.03) | 0.04 (0.02-0.06) | 0.01 (0.00-0.03) | 0.01 (0.00-0.02) | 0.04 (0.03-0.06) | 0.08 (0.06-0.10) | 0.01 (0.01-0.03) |
| Mixed | 0.09 (0.06-0.13) | 0.19 (0.15-0.25) | 0.27 (0.21-0.34) | 0.08 (0.05-0.13) | 0.10 (0.08-0.13) | 0.18 (0.15-0.22) | 0.34 (0.29-0.39) | 0.15 (0.11-0.20) | 0.10 (0.08-0.12) | 0.19 (0.16-0.22) | 0.32 (0.28-0.36) | 0.13 (0.10-0.16) |
| Unknown infection | 1.56 (1.43-1.70) | 2.79 (2.60-2.98) | 3.34 (3.11-3.57) | 1.50 (1.32-1.69) | 1.63 (1.53-1.73) | 1.87 (1.77-1.98) | 2.62 (2.49-2.77) | 1.70 (1.55-1.86) | 1.61 (1.53-1.69) | 2.17 (2.07-2.27) | 2.86 (2.74-2.98) | 1.61 (1.50-1.73) |

Provided values represent cumulative incidence (95%CI).

**Table-S2. Causes of deaths by GVHD, transplant period and calendar period in allo-HSCT patients**

|  | Year of last transplant | | | | | | | | Total | | | |
| --- | --- | --- | --- | --- | --- | --- | --- | --- | --- | --- | --- | --- |
|  | 1980-2001 | | | | 2002-2015 | | | |  |  |  |  |
| **Allogeneic** | N=31128 | | | | N=64661 | | | | N=95789 | | | |
|  | 30 days | 100 days | 1 year | 5 years | 30 days | 100 days | 1 year | 5 years | 30 days | 100 days | 1 year | 5 years |
| **Main cause of death** |  | alive at day 30 | alive at day 100 | alive at 1 year |  | alive at day 30 | alive at day 100 | alive at 1 year |  | alive at day 30 | alive at day 100 | alive at 1 year |
| **Relapse all Pts** | 0.26 (0.21-0.32) | 2.12 (1.96-2.29) | 9.85 (9.47-10.23) | 10.71 (10.25-11.18) | 0.38 (0.33-0.43) | 2.96 (2.83-3.10) | 13.18 (12.88-13.48) | 13.26 (12.85-13.67) | 0.34 (0.30-0.38) | 2.69 (2.58-2.80) | 12.08 (11.84-12.32) | 12.29 (11.98-12.60) |
| With GvHD | - | 1.54 (1.23-1.91) | 7.91 (7.19-8.67) | 9.02 (8.36-9.70) | - | 2.47 (2.07-2.91) | 10.89 (10.16-11.64) | 11.63 (11.09-12.18) | - | 2.01 (1.75-2.29) | 9.62 (9.10-10.16) | 10.75 (10.33-11.18) |
| No GvHD | - | 2.24 (2.03-2.46) | 11.00 (10.50-11.52) | 10.69 (10.03-11.38) | - | 3.14 (2.98-3.31) | 13.99 (13.61-14.37) | 14.19 (13.57-14.81) | - | 2.87 (2.74-3.01) | 13.07 (12.77-13.38) | 12.83 (12.37-13.29) |
|  |  |  |  |  |  |  |  |  |  |  |  |  |
| **GvHD all Pts** | 0.66 (0.57-0.75) | 5.60 (5.34-5.87) | 5.21 (4.94-5.50) | 2.95 (2.71-3.22) | 0.20 (0.17-0.24) | 2.49 (2.37-2.61) | 4.28 (4.10-4.46) | 3.85 (3.62-4.08) | 0.35 (0.31-0.39) | 3.50 (3.38-3.62) | 4.58 (4.43-4.74) | 3.50 (3.33-3.67) |
| With GvHD | - | 15.42 (14.45-16.42) | 12.32 (11.43-13.24) | 4.94 (4.45-5.46) | - | 7.21 (6.54-7.93) | 8.98 (8.32-9.68) | 5.67 (5.36-6.00) | - | 11.26 (10.66-11.87) | 10.39 (9.85-10.95) | 6.08 (5.68-6.50) |
| No GvHD | - | 1.46 (1.29-1.64) | 1.65 (1.45-1.87) | 1.55 (1.30-1.84) | - | 0.59 (0.52-0.66) | 2.02 (1.87-2.18) | 1.59 (1.38-1.83) | - | 0.85 (0.78-0.92) | 1.90 (1.78-2.03) | 1.57 (1.40-1.75) |
|  |  |  |  |  |  |  |  |  |  |  |  |  |
| **Infection all Pts** | 2.73 (2.56-2.92) | 5.00 (4.76-5.26) | 6.45 (6.14-6.76) | 2.89 (2.65-3.15) | 2.25 (2.13-2.36) | 2.85 (2.72-2.98) | 4.34 (4.17-4.53) | 2.85 (2.66-3.05) | 2.41 (2.31-2.50) | 3.55 (3.43-3.67) | 5.03 (4.88-5.20) | 2.86 (2.71-3.02) |
| With GvHD | - | 5.95 (5.32-6.62) | 9.75 (8.95-10.58) | 3.69 (3.26-4.14) | - | 2.25 (1.88-2.68) | 6.03 (5.49-6.62) | 3.47 (3.17-3.78) | - | 4.07 (3.71-4.47) | 7.61 (7.14-8.10) | 3.54 (3.29-3.80) |
| No GvHD | - | 4.70 (4.40-5.02) | 5.25 (4.90-5.62) | 2.05 (1.76-2.38) | - | 2.83 (2.67-2.99) | 3.66 (3.46-3.87) | 2.16 (1.92-2.43) | - | 3.38 (3.24-3.53) | 4.15 (3.97-4.33) | 2.11 (1.92-2.32) |
|  |  |  |  |  |  |  |  |  |  |  |  |  |
| **Other causes all Pts** | 2.26 (2.10-2.43) | 2.90 (2.71-3.10) | 3.11 (2.90-3.33) | 2.54 (2.32-2.79) | 1.25 (1.17-1.34) | 1.78 (1.67-1.88) | 2.79 (2.65-2.94) | 3.45 (3.23-3.68) | 1.58 (1.50-1.66) | 2.14 (2.05-2.24) | 2.90 (2.77-3.02) | 3.09 (2.93-3.26) |
| With GvHD | - | 3.04 (2.60-3.54) | 4.28 (3.75-4.86) | 3.08 (2.73-3.46) | - | 1.95 (1.60-2.35) | 3.42 (3.00-3.87) | 3.89 (3.59-4.21) | - | 2.49 (2.20-2.80) | 3.78 (3.45-4.13) | 3.74 (3.48-4.01) |
| No GvHD | - | 2.61 (2.39-2.85) | 2.27 (2.03-2.52) | 1.75 (1.44-2.09) | - | 1.62 (1.50-1.74) | 2.43 (2.27-2.61) | 2.50 (2.20-2.83) | - | 1.91 (1.81-2.03) | 2.38 (2.25-2.52) | 2.35 (2.15-2.57) |
|  |  |  |  |  |  |  |  |  |  |  |  |  |
| **Unknown all Pts** | 0.22 (0.17-0.28) | 0.50 (0.42-0.59) | 0.85 (0.74-0.97) | 1.04 (0.89-1.20) | 0.06 (0.05-0.08) | 0.15 (0.12-0.19) | 0.54 (0.48-0.61) | 0.90 (0.79-1.03) | 0.11 (0.09-0.14) | 0.27 (0.23-0.30) | 0.64 (0.59-0.70) | 0.95 (0.86-1.05) |
| With GvHD | - | 0.39 (0.25-0.59) | 0.71 (0.51-0.98) | 1.07 (0.85-1.34) | - | 0.06 (0.02-0.16) | 0.38 (0.26-0.55) | 1.04 (0.87-1.23) | - | 0.22 (0.15-0.33) | 0.52 (0.41-0.67) | 1.05 (0.92-1.20) |
| No GvHD | - | 0.34 (0.26-0.43) | 0.49 (0.39-0.62) | 0.57 (0.42-0.76) | - | 0.12 (0.09-0.15) | 0.43 (0.36-0.51) | 0.46 (0.35-0.61) | - | 0.18 (0.15-0.22) | 0.45 (0.39-0.51) | 0.50 (0.41-0.61) |
|  |  |  |  |  |  |  |  |  |  |  |  |  |
| **Total mortality all Pts** | 6.13 (5.87-6.40) | 16.12 (15.70-16.55) | 25.47 (24.92-26.02) | 20.14 (19.54-20.74) | 4.14 (3.98-4.29) | 10.23 (9.99-10.47) | 25.14 (24.75-25.52) | 24.31 (23.79-24.83) | 4.79 (4.65-4.92) | 12.14 (11.93-12.36) | 25.23 (24.92-25.55) | 22.69 (22.30-23.08) |
| With GvHD | - | 26.34 (25.14-27.55) | 34.97 (33.65-36.28) | 21.86 (20.90-22.84) | - | 13.94 (13.02-14.89) | 29.70 (28.62-30.79) | 26.26 (25.50-27.03) | - | 20.05 (19.29-20.83) | 31.93 (31.09-32.77) | 24.75 (24.15-25.35) |
| No GvHD | - | 11.35 (10.89-11.81) | 20.67 (20.02-21.33) | 16.84 (16.03-17.67) | - | 8.29 (8.03-8.55) | 22.53 (22.08-22.99) | 21.02 (20.29-21.76) | - | 9.20 (8.97-9.43) | 21.95 (21.57-22.32) | 19.36 (18.82-19.91) |

Provided values represent cumulative incidence (95%CI).

**Table-S3A. Causes of deaths by calendar period**

|  | Year of last transplant | | | | | | | | Total | | | |
| --- | --- | --- | --- | --- | --- | --- | --- | --- | --- | --- | --- | --- |
|  | 1980-2001 | | | | 2002-2015 | | | |  |  |  |  |
|  | N=42997 | | | | N=71494 | | | | N=114491 | | | |
| Cumulative incidence of death due to |  | alive at day 30 | alive at day 100 | alive at 1 year |  | alive at day 30 | alive at day 100 | alive at 1 year |  | alive at day 30 | alive at day 100 | alive at 1 year |
|  | Cumulative incidence at | | | | Cumulative incidence at | | | | Cumulative incidence at | | | |
|  | 30 days | 100 days | 1 year | 5 years | 30 days | 100 days | 1 year | 5 years | 30 days | 100 days | 1 year | 5 years |
| Relapse | 0.27  (0.23-0.33) | 2.41  (2.26-2.56) | 13.01  (12.66-13.37) | 14.37  (13.93-14.82) | 0.37  (0.33-0.42) | 2.88  (2.76-3.01) | 13.69  (13.40-13.98) | 13.76  (13.37-14.15) | 0.34  (0.30-0.37) | 2.70  (2.61-2.80) | 13.43  (13.21-13.66) | 14.03  (13.74-14.32) |
| GvHD | 0.49  (0.43-0.56) | 4.05  (3.86-4.25) | 3.69  (3.49-3.89) | 2.15  (1.97-2.34) | 0.18  (0.15-0.21) | 2.25  (2.14-2.37) | 3.86  (3.70-4.02) | 3.46  (3.25-3.67) | 0.30 (0.27.0.33) | 2.93  (2.83-3.03) | 3.79  (3.66-3.92) | 2.88  (2.74-3.02) |
| Infection | 2.44  (2.30-2.59) | 4.18  (3.99-4.38) | 5.59  (5.35-5.83) | 2.89  (2.69-3.11) | 2.14  (2.03-2.25) | 2.65  (2.53-2.77) | 4.10  (3.94-4.27) | 2.72  (2.54-2.91) | 2.25  (2.17-2.34) | 3.23  (3.12-3.33) | 4.67  (4.53-4.81) | 2.80  (2.66-2.94) |
| Other causes | 1.95  (1.82-2.09) | 2.59  (2.44-2.75) | 2.94  (2.77-3.13) | 2.69  (2.49-2.90) | 1.19  (1.11-1.27) | 1.67  (1.58-1.77) | 2.72  (2.59-2.86) | 3.47  (3.27-3.69) | 1.48  (1.41-1.55) | 2.02  (1.94-2.10) | 2.81  (2.70-2.92) | 3.12  (2.97-3.27) |
| Unknown cause | 0.19  (0.15-0.23) | 0.44  (0.38-0.51) | 0.83  (0.74-0.93) | 1.17  (1.04-1.31) | 0.07  (0.05-0.09) | 0.15  (0.12-0.18) | 0.56  (0.50-0.63) | 0.93  (0.82-1.05) | 0.11  (0.10-0.13) | 0.26  (0.23-0.29) | 0.66  (0.61-0.72) | 1.04  (0.95-1.13) |
| Total mortality | 5.35  (5.14-5.57) | 13.67  (13.34-14.01) | 26.06  (25.59-26.52) | 23.27  (22.73-23.81) | 3.95  (3.81-4.09) | 9.61  (9.39-9.83) | 24.93  (24.56-25.30) | 24.34  (23.85-24.83) | 4.48  (4.36-4.60) | 11.14  (10.95-11.33) | 25.36  (25.08-25.65) | 23.86  (23.49-24.22) |

**Tables-S3B. Causes of deaths by transplant period and calendar period**

|  |  |  |  |  |  | Cum Inc (95% CI) | | | |  |
| --- | --- | --- | --- | --- | --- | --- | --- | --- | --- | --- |
| relapse | group | event | competing | Censored | Patients | 30 days | 100 days | 1 year | 5 years | Gray test |
|  | 1980-2001 | 9627 | 14943 | 18427 | 42997 | 0.27 (0.23-0.33) | 2.55 (2.40-2.70) | 13.18 (12.86-13.51) | 21.86 (21.46-22.27) | <0.0001 |
|  | 2002-2015 | 14149 | 16949 | 40396 | 71494 | 0.37 (0.33-0.42) | 3.14 (3.02-3.28) | 15.03 (14.75-15.31) | 23.99 (23.64-24.35) |  |
|  | Total | 23776 | 31892 | 58823 | 114491 | 0.34 (0.30-0.37) | 2.92 (2.82-3.02) | 14.32 (14.11-14.53) | 23.21 (22.94-23.48) |  |
|  |  |  |  |  |  |  |  |  |  |  |
| gvhd | group | event | competing | Censored | Patients | 30 days | 100 days | 1 year | 5 years | Gray test |
|  | 1980-2001 | 3797 | 20773 | 18427 | 42997 | 0.49 (0.43-0.56) | 4.33 (4.14-4.52) | 7.34 (7.10-7.59) | 8.64 (8.37-8.91) | <0.0001 |
|  | 2002-2015 | 4906 | 26192 | 40396 | 71494 | 0.18 (0.15-0.21) | 2.35 (2.23-2.46) | 5.70 (5.52-5.88) | 7.95 (7.73-8.17) |  |
|  | Total | 8703 | 46965 | 58823 | 114491 | 0.30 (0.27-0.33) | 3.10 (3.00-3.20) | 6.31 (6.17-6.46) | 8.14 (7.97-8.31) |  |
|  |  |  |  |  |  |  |  |  |  |  |
| infection | group | event | competing | Censored | Patients | 30 days | 100 days | 1 year | 5 years | Gray test |
|  | 1980-2001 | 5486 | 19084 | 18427 | 42997 | 2.44 (2.30-2.59) | 6.40 (6.17-6.64) | 10.96 (10.67-11.26) | 12.71 (12.39-13.03) | <0.0001 |
|  | 2002-2015 | 6455 | 24643 | 40396 | 71494 | 2.14 (2.03-2.25) | 4.68 (4.53-4.84) | 8.24 (8.04-8.45) | 10.02 (9.78-10.26) |  |
|  | Total | 11941 | 43727 | 58823 | 114491 | 2.25 (2.17-2.34) | 5.34 (5.21-5.47) | 9.30 (9.13-9.48) | 11.07 (10.88-11.26) |  |
|  |  |  |  |  |  |  |  |  |  |  |
| other causes | group | event | competing | Censored | Patients | 30 days | 100 days | 1 year | 5 years | Gray test |
|  | 1980-2001 | 4364 | 20206 | 18427 | 42997 | 1.95 (1.82-2.09) | 4.41 (4.21-4.60) | 6.81 (6.57-7.05) | 8.43 (8.17-8.70) | <0.0001 |
|  | 2002-2015 | 4764 | 26334 | 40396 | 71494 | 1.19 (1.11-1.27) | 2.79 (2.67-2.92) | 5.16 (4.99-5.33) | 7.42 (7.21-7.64) |  |
|  | Total | 9128 | 46540 | 58823 | 114491 | 1.48 (1.41-1.55) | 3.40 (3.30-3.51) | 5.79 (5.65-5.93) | 7.76 (7.60-7.93) |  |
|  |  |  |  |  |  |  |  |  |  |  |
| unknown | group | event | competing | Censored | Patients | 30 days | 100 days | 1 year | 5 years | Gray test |
|  | 1980-2001 | 1296 | 23274 | 18427 | 42997 | 0.19 (0.15-0.23) | 0.61 (0.54-0.68) | 1.28 (1.18-1.40) | 1.99 (1.86-2.13) | <0.0001 |
|  | 2002-2015 | 824 | 30274 | 40396 | 71494 | 0.07 (0.05-0.09) | 0.21 (0.18-0.25) | 0.70 (0.64-0.76) | 1.30 (1.21-1.40) |  |
|  | Total | 2120 | 53548 | 58823 | 114491 | 0.11 (0.10-0.13) | 0.36 (0.33-0.40) | 0.93 (0.87-0.98) | 1.58 (1.50-1.66) |  |
|  |  |  |  |  |  |  |  |  |  |  |
| all deaths | group | event | competing | Censored | Patients | 30 days | 100 days | 1 year | 5 years | Gray test |
|  | 1980-2001 | 24570 | 0 | 18427 | 42997 | 5.35 (5.14-5.57) | 18.29 (17.93-18.66) | 39.58 (39.12-40.05) | 53.64 (53.16-54.12) | <0.0001 |
|  | 2002-2015 | 31098 | 0 | 40396 | 71494 | 3.95 (3.81-4.09) | 13.18 (12.93-13.43) | 34.82 (34.45-35.19) | 50.68 (50.26-51.11) |  |
|  | Total | 55668 | 0 | 58823 | 114491 | 4.48 (4.36-4.60) | 15.12 (14.91-15.33) | 36.65 (36.36-36.94) | 51.76 (51.44-52.08) |  |

**Tables-S4A. Causes of deaths by main cause, transplant period and calendar period in total cohort**

All patients

1. Relapse

| group | timepoint | event | competing | Censored | Patients | Cum Inc (95% CI) | Gray test |
| --- | --- | --- | --- | --- | --- | --- | --- |
| 1980-2001 | 30 days | 117 | 2175 | 40705 | 42997 | 0.27 (0.23-0.33) | 0.0044 |
| 2002-2015 | 30 days | 265 | 2532 | 68697 | 71494 | 0.37 (0.33-0.42) |  |
| Total | 30 days | 382 | 4707 | 109402 | 114491 | 0.34 (0.30-0.37) |  |
| Alive at 30 days | |  |  |  |  |  |  |
| 1980-2001 | 100 days | 969 | 4544 | 34967 | 40480 | 2.41 (2.26-2.56) | <0.0001 |
| 2002-2015 | 100 days | 1916 | 4487 | 61220 | 67623 | 2.88 (2.76-3.01) |  |
| Total | 100 days | 2885 | 9031 | 96187 | 108103 | 2.70 (2.61-2.80) |  |
| Alive at 100 days | |  |  |  |  |  |  |
| 1980-2001 | 1 year | 4435 | 4460 | 25724 | 34619 | 13.01 (12.66-13.37) | 0.0021 |
| 2002-2015 | 1 year | 7366 | 6083 | 45160 | 58609 | 13.69 (13.40-13.98) |  |
| Total | 1 year | 11801 | 10543 | 70884 | 93228 | 13.43 (13.21-13.66) |  |
| Alive at 1 year | |  |  |  |  |  |  |
| 1980-2001 | 5 years | 3447 | 2125 | 19224 | 24796 | 14.37 (13.93-14.82) | 0.0499 |
| 2002-2015 | 5 years | 4303 | 3224 | 29960 | 37487 | 13.76 (13.37-14.15) |  |
| Total | 5 years | 7750 | 5349 | 49184 | 62283 | 14.03 (13.74-14.32) |  |

1. GvHD

| group | timepoint | event | competing | Censored | Patients | Cum Inc (95% CI) | Gray test |
| --- | --- | --- | --- | --- | --- | --- | --- |
| 1980-2001 | 30 days | 204 | 2088 | 40705 | 42997 | 0.48 (0.42-0.55) | <0.0001 |
| 2002-2015 | 30 days | 127 | 2670 | 68697 | 71494 | 0.18 (0.15-0.21) |  |
| Total | 30 days | 331 | 4758 | 109402 | 114491 | 0.29 (0.26-0.32) |  |
| Alive at 30 days | |  |  |  |  |  |  |
| 1980-2001 | 100 days | 1627 | 3886 | 34967 | 40480 | 4.03 (3.85-4.23) | <0.0001 |
| 2002-2015 | 100 days | 1501 | 4902 | 61220 | 67623 | 2.25 (2.14-2.37) |  |
| Total | 100 days | 3128 | 8788 | 96187 | 108103 | 2.92 (2.82-3.03) |  |
| Alive at 100 days | |  |  |  |  |  |  |
| 1980-2001 | 1 year | 1254 | 7641 | 25724 | 34619 | 3.66 (3.47-3.87) | 0.1998 |
| 2002-2015 | 1 year | 2089 | 11360 | 45160 | 58609 | 3.85 (3.69-4.02) |  |
| Total | 1 year | 3343 | 19001 | 70884 | 93228 | 3.78 (3.65-3.90) |  |
| Alive at 1 year | |  |  |  |  |  |  |
| 1980-2001 | 5 years | 504 | 5068 | 19224 | 24796 | 2.11 (1.93-2.30) | <0.0001 |
| 2002-2015 | 5 years | 1055 | 6472 | 29960 | 37487 | 3.44 (3.24-3.65) |  |
| Total | 5 years | 1559 | 11540 | 49184 | 62283 | 2.85 (2.71-2.99) |  |

1. Infection - Total

| group | timepoint | event | competing | Censored | Patients | Cum Inc (95% CI) | Gray test |
| --- | --- | --- | --- | --- | --- | --- | --- |
| 1980-2001 | 30 days | 1047 | 1245 | 40705 | 42997 | 2.44 (2.30-2.59) | 0.0009 |
| 2002-2015 | 30 days | 1515 | 1282 | 68697 | 71494 | 2.14 (2.03-2.25) |  |
| Total | 30 days | 2562 | 2527 | 109402 | 114491 | 2.25 (2.17-2.34) |  |
| Alive at 30 days | |  |  |  |  |  |  |
| 1980-2001 | 100 days | 1686 | 3827 | 34967 | 40480 | 4.18 (3.99-4.38) | <0.0001 |
| 2002-2015 | 100 days | 1769 | 4634 | 61220 | 67623 | 2.65 (2.53-2.77) |  |
| Total | 100 days | 3455 | 8461 | 96187 | 108103 | 3.23 (3.12-3.33) |  |
| Alive at 100 days | |  |  |  |  |  |  |
| 1980-2001 | 1 year | 1909 | 6986 | 25724 | 34619 | 5.59 (5.35-5.83) | <0.0001 |
| 2002-2015 | 1 year | 2220 | 11229 | 45160 | 58609 | 4.10 (3.94-4.27) |  |
| Total | 1 year | 4129 | 18215 | 70884 | 93228 | 4.67 (4.53-4.81) |  |
| Alive at 1 year | |  |  |  |  |  |  |
| 1980-2001 | 5 years | 695 | 4877 | 19224 | 24796 | 2.89 (2.69-3.11) | 0.2344 |
| 2002-2015 | 5 years | 860 | 6667 | 29960 | 37487 | 2.72 (2.54-2.91) |  |
| Total | 5 years | 1555 | 11544 | 49184 | 62283 | 2.80 (2.66-2.94) |  |

1. Other causes

| group | timepoint | event | competing | Censored | Patients | Cum Inc (95% CI) | Gray test |
| --- | --- | --- | --- | --- | --- | --- | --- |
| 1980-2001 | 30 days | 843 | 1449 | 40705 | 42997 | 1.97 (1.84-2.10) | <0.0001 |
| 2002-2015 | 30 days | 842 | 1955 | 68697 | 71494 | 1.19 (1.11-1.27) |  |
| Total | 30 days | 1685 | 3404 | 109402 | 114491 | 1.48 (1.41-1.55) |  |
| Alive at 30 days | |  |  |  |  |  |  |
| 1980-2001 | 100 days | 1053 | 4460 | 34967 | 40480 | 2.61 (2.46-2.77) | <0.0001 |
| 2002-2015 | 100 days | 1118 | 5285 | 61220 | 67623 | 1.67 (1.58-1.77) |  |
| Total | 100 days | 2171 | 9745 | 96187 | 108103 | 2.03 (1.94-2.11) |  |
| Alive at 100 days | |  |  |  |  |  |  |
| 1980-2001 | 1 year | 1014 | 7881 | 25724 | 34619 | 2.97 (2.79-3.15) | 0.0288 |
| 2002-2015 | 1 year | 1472 | 11977 | 45160 | 58609 | 2.73 (2.59-2.87) |  |
| Total | 1 year | 2486 | 19858 | 70884 | 93228 | 2.82 (2.71-2.93) |  |
| Alive at 1 year | |  |  |  |  |  |  |
| 1980-2001 | 5 years | 649 | 4923 | 19224 | 24796 | 2.73 (2.53-2.94) | <0.0001 |
| 2002-2015 | 5 years | 1037 | 6490 | 29960 | 37487 | 3.49 (3.28-3.71) |  |
| Total | 5 years | 1686 | 11413 | 49184 | 62283 | 3.14 (3.00-3.29) |  |

1. Cause of death unknown

| group | timepoint | event | competing | Censored | Patients | Cum Inc (95% CI) | Gray test |
| --- | --- | --- | --- | --- | --- | --- | --- |
| 1980-2001 | 30 days | 81 | 2211 | 40705 | 42997 | 0.19 (0.15-0.23) | <0.0001 |
| 2002-2015 | 30 days | 48 | 2749 | 68697 | 71494 | 0.07 (0.05-0.09) |  |
| Total | 30 days | 129 | 4960 | 109402 | 114491 | 0.11 (0.10-0.13) |  |
| Alive at 30 days | |  |  |  |  |  |  |
| 1980-2001 | 100 days | 178 | 5335 | 34967 | 40480 | 0.44 (0.38-0.51) | <0.0001 |
| 2002-2015 | 100 days | 99 | 6304 | 61220 | 67623 | 0.15 (0.12-0.18) |  |
| Total | 100 days | 277 | 11639 | 96187 | 108103 | 0.26 (0.23-0.29) |  |
| Alive at 100 days | |  |  |  |  |  |  |
| 1980-2001 | 1 year | 283 | 8612 | 25724 | 34619 | 0.83 (0.74-0.93) | <0.0001 |
| 2002-2015 | 1 year | 302 | 13147 | 45160 | 58609 | 0.56 (0.50-0.63) |  |
| Total | 1 year | 585 | 21759 | 70884 | 93228 | 0.66 (0.61-0.72) |  |
| Alive at 1 year | |  |  |  |  |  |  |
| 1980-2001 | 5 years | 277 | 5295 | 19224 | 24796 | 1.17 (1.04-1.31) | 0.0067 |
| 2002-2015 | 5 years | 272 | 7255 | 29960 | 37487 | 0.93 (0.82-1.05) |  |
| Total | 5 years | 549 | 12550 | 49184 | 62283 | 1.04 (0.95-1.13) |  |

1. All causes of death

| group | timepoint | event | competing | Censored | Patients | Cum Inc (95% CI) | Gray test |
| --- | --- | --- | --- | --- | --- | --- | --- |
| 1980-2001 | 30 days | 2292 | 0 | 40705 | 42997 | 5.35 (5.14-5.57) | <0.0001 |
| 2002-2015 | 30 days | 2797 | 0 | 68697 | 71494 | 3.95 (3.81-4.09) |  |
| Total | 30 days | 5089 | 0 | 109402 | 114491 | 4.48 (4.36-4.60) |  |
| Alive at 30 days | |  |  |  |  |  |  |
| 1980-2001 | 100 days | 5513 | 0 | 34967 | 40480 | 13.67 (13.34-14.01) | <0.0001 |
| 2002-2015 | 100 days | 6403 | 0 | 61220 | 67623 | 9.61 (9.39-9.83) |  |
| Total | 100 days | 11916 | 0 | 96187 | 108103 | 11.14 (10.95-11.33) |  |
| Alive at 100 days | |  |  |  |  |  |  |
| 1980-2001 | 1 year | 8895 | 0 | 25724 | 34619 | 26.06 (25.59-26.52) | <0.0001 |
| 2002-2015 | 1 year | 13449 | 0 | 45160 | 58609 | 24.93 (24.56-25.30) |  |
| Total | 1 year | 22344 | 0 | 70884 | 93228 | 25.36 (25.08-25.65) |  |
| Alive at 1 year | |  |  |  |  |  |  |
| 1980-2001 | 5 years | 5572 | 0 | 19224 | 24796 | 23.27 (22.73-23.81) | 0.0051 |
| 2002-2015 | 5 years | 7527 | 0 | 29960 | 37487 | 24.34 (23.85-24.83) |  |
| Total | 5 years | 13099 | 0 | 49184 | 62283 | 23.86 (23.49-24.22) |  |

**Tables-S4B. Causes of infectious deaths by etiology, transplant period and calendar period in total cohort**

All patients

1. Bacterial

| group | timepoint | event | competing | Censored | Patients | Cum Inc (95% CI) | Gray test |
| --- | --- | --- | --- | --- | --- | --- | --- |
| 1980-2001 | 30 days | 224 | 2068 | 40705 | 42997 | 0.52 (0.46-0.60) | <0.0001 |
| 2002-2015 | 30 days | 230 | 2567 | 68697 | 71494 | 0.32 (0.29-0.37) |  |
| Total | 30 days | 454 | 4635 | 109402 | 114491 | 0.40 (0.36-0.44) |  |
| Alive at 30 days | |  |  |  |  |  |  |
| 1980-2001 | 100 days | 183 | 5330 | 34967 | 40480 | 0.45 (0.39-0.52) | <0.0001 |
| 2002-2015 | 100 days | 176 | 6227 | 61220 | 67623 | 0.26 (0.23-0.31) |  |
| Total | 100 days | 359 | 11557 | 96187 | 108103 | 0.34 (0.30-0.37) |  |
| Alive at 100 days | |  |  |  |  |  |  |
| 1980-2001 | 1 year | 307 | 8588 | 25724 | 34619 | 0.90 (0.80-1.00) | <0.0001 |
| 2002-2015 | 1 year | 241 | 13208 | 45160 | 58609 | 0.45 (0.39-0.51) |  |
| Total | 1 year | 548 | 21796 | 70884 | 93228 | 0.62 (0.57-0.68) |  |
| Alive at 1 year | |  |  |  |  |  |  |
| 1980-2001 | 5 years | 181 | 5391 | 19224 | 24796 | 0.75 (0.65-0.87) | 0.0003 |
| 2002-2015 | 5 years | 160 | 7367 | 29960 | 37487 | 0.51 (0.44-0.60) |  |
| Total | 5 years | 341 | 12758 | 49184 | 62283 | 0.62 (0.55-0.68) |  |

1. Viral

| group | timepoint | event | competing | Censored | Patients | Cum Inc (95% CI) | Gray test |
| --- | --- | --- | --- | --- | --- | --- | --- |
| 1980-2001 | 30 days | 39 | 2253 | 40705 | 42997 | 0.09 (0.07-0.12) | 0.0413 |
| 2002-2015 | 30 days | 41 | 2756 | 68697 | 71494 | 0.06 (0.04-0.08) |  |
| Total | 30 days | 80 | 5009 | 109402 | 114491 | 0.07 (0.06-0.09) |  |
| Alive at 30 days | |  |  |  |  |  |  |
| 1980-2001 | 100 days | 233 | 5280 | 34967 | 40480 | 0.58 (0.51-0.66) | <0.0001 |
| 2002-2015 | 100 days | 201 | 6202 | 61220 | 67623 | 0.30 (0.26-0.35) |  |
| Total | 100 days | 434 | 11482 | 96187 | 108103 | 0.41 (0.37-0.45) |  |
| Alive at 100 days | |  |  |  |  |  |  |
| 1980-2001 | 1 year | 214 | 8681 | 25724 | 34619 | 0.63 (0.55-0.71) | 0.0002 |
| 2002-2015 | 1 year | 240 | 13209 | 45160 | 58609 | 0.44 (0.39-0.50) |  |
| Total | 1 year | 454 | 21890 | 70884 | 93228 | 0.51 (0.47-0.56) |  |
| Alive at 1 year | |  |  |  |  |  |  |
| 1980-2001 | 5 years | 46 | 5526 | 19224 | 24796 | 0.19 (0.14-0.26) | 0.6006 |
| 2002-2015 | 5 years | 67 | 7460 | 29960 | 37487 | 0.21 (0.16-0.26) |  |
| Total | 5 years | 113 | 12986 | 49184 | 62283 | 0.20 (0.17-0.24) |  |

1. Fungal

| group | timepoint | event | competing | Censored | Patients | Cum Inc (95% CI) | Gray test |
| --- | --- | --- | --- | --- | --- | --- | --- |
| 1980-2001 | 30 days | 159 | 2133 | 40705 | 42997 | 0.37 (0.32-0.43) | <0.0001 |
| 2002-2015 | 30 days | 83 | 2714 | 68697 | 71494 | 0.12 (0.09-0.15) |  |
| Total | 30 days | 242 | 4847 | 109402 | 114491 | 0.21 (0.19-0.24) |  |
| Alive at 30 days | |  |  |  |  |  |  |
| 1980-2001 | 100 days | 237 | 5276 | 34967 | 40480 | 0.59 (0.52-0.67) | <0.0001 |
| 2002-2015 | 100 days | 108 | 6295 | 61220 | 67623 | 0.16 (0.13-0.20) |  |
| Total | 100 days | 345 | 11571 | 96187 | 108103 | 0.32 (0.29-0.36) |  |
| Alive at 100 days | |  |  |  |  |  |  |
| 1980-2001 | 1 year | 288 | 8607 | 25724 | 34619 | 0.84 (0.75-0.94) | <0.0001 |
| 2002-2015 | 1 year | 204 | 13245 | 45160 | 58609 | 0.38 (0.33-0.43) |  |
| Total | 1 year | 492 | 21852 | 70884 | 93228 | 0.56 (0.51-0.61) |  |
| Alive at 1 year | |  |  |  |  |  |  |
| 1980-2001 | 5 years | 90 | 5482 | 19224 | 24796 | 0.37 (0.30-0.46) | 0.0056 |
| 2002-2015 | 5 years | 79 | 7448 | 29960 | 37487 | 0.24 (0.19-0.30) |  |
| Total | 5 years | 169 | 12930 | 49184 | 62283 | 0.30 (0.26-0.35) |  |

1. Parasitic

| group | timepoint | event | competing | Censored | Patients | Cum Inc (95% CI) | Gray test |
| --- | --- | --- | --- | --- | --- | --- | --- |
| 1980-2001 | 30 days | 5 | 2287 | 40705 | 42997 | 0.01 (0.00-0.03) | 0.1480 |
| 2002-2015 | 30 days | 3 | 2794 | 68697 | 71494 | 0.00 (0.00-0.01) |  |
| Total | 30 days | 8 | 5081 | 109402 | 114491 | 0.01 (0.00-0.01) |  |
| Alive at 30 days | |  |  |  |  |  |  |
| 1980-2001 | 100 days | 30 | 5483 | 34967 | 40480 | 0.07 (0.05-0.11) | <0.0001 |
| 2002-2015 | 100 days | 12 | 6391 | 61220 | 67623 | 0.02 (0.01-0.03) |  |
| Total | 100 days | 42 | 11874 | 96187 | 108103 | 0.04 (0.03-0.05) |  |
| Alive at 100 days | |  |  |  |  |  |  |
| 1980-2001 | 1 year | 40 | 8855 | 25724 | 34619 | 0.12 (0.09-0.16) | <0.0001 |
| 2002-2015 | 1 year | 20 | 13429 | 45160 | 58609 | 0.04 (0.02-0.06) |  |
| Total | 1 year | 60 | 22284 | 70884 | 93228 | 0.07 (0.05-0.09) |  |
| Alive at 1 year | |  |  |  |  |  |  |
| 1980-2001 | 5 years | 3 | 5569 | 19224 | 24796 | 0.01 (0.00-0.04) | 0.7056 |
| 2002-2015 | 5 years | 3 | 7524 | 29960 | 37487 | 0.01 (0.00-0.03) |  |
| Total | 5 years | 6 | 13093 | 49184 | 62283 | 0.01 (0.00-0.02) |  |

1. Mixed

| group | timepoint | event | competing | Censored | Patients | Cum Inc (95% CI) | Gray test |
| --- | --- | --- | --- | --- | --- | --- | --- |
| 1980-2001 | 30 days | 40 | 2252 | 40705 | 42997 | 0.09 (0.07-0.13) | 0.9913 |
| 2002-2015 | 30 days | 66 | 2731 | 68697 | 71494 | 0.09 (0.07-0.12) |  |
| Total | 30 days | 106 | 4983 | 109402 | 114491 | 0.09 (0.08-0.11) |  |
| Alive at 30 days | |  |  |  |  |  |  |
| 1980-2001 | 100 days | 72 | 5441 | 34967 | 40480 | 0.18 (0.14-0.22) | 0.7284 |
| 2002-2015 | 100 days | 113 | 6290 | 61220 | 67623 | 0.17 (0.14-0.20) |  |
| Total | 100 days | 185 | 11731 | 96187 | 108103 | 0.17 (0.15-0.20) |  |
| Alive at 100 days | |  |  |  |  |  |  |
| 1980-2001 | 1 year | 89 | 8806 | 25724 | 34619 | 0.26 (0.21-0.32) | 0.2257 |
| 2002-2015 | 1 year | 166 | 13283 | 45160 | 58609 | 0.31 (0.26-0.36) |  |
| Total | 1 year | 255 | 22089 | 70884 | 93228 | 0.29 (0.25-0.33) |  |
| Alive at 1 year | |  |  |  |  |  |  |
| 1980-2001 | 5 years | 21 | 5551 | 19224 | 24796 | 0.09 (0.06-0.13) | 0.0388 |
| 2002-2015 | 5 years | 49 | 7478 | 29960 | 37487 | 0.15 (0.11-0.20) |  |
| Total | 5 years | 70 | 13029 | 49184 | 62283 | 0.12 (0.10-0.15) |  |

1. Infection type unknown

| group | timepoint | event | competing | Censored | Patients | Cum Inc (95% CI) | Gray test |
| --- | --- | --- | --- | --- | --- | --- | --- |
| 1980-2001 | 30 days | 580 | 1712 | 40705 | 42997 | 1.35 (1.25-1.47) | 0.0103 |
| 2002-2015 | 30 days | 1092 | 1705 | 68697 | 71494 | 1.54 (1.45-1.63) |  |
| Total | 30 days | 1672 | 3417 | 109402 | 114491 | 1.47 (1.40-1.54) |  |
| Alive at 30 days | |  |  |  |  |  |  |
| 1980-2001 | 100 days | 931 | 4582 | 34967 | 40480 | 2.31 (2.16-2.46) | <0.0001 |
| 2002-2015 | 100 days | 1159 | 5244 | 61220 | 67623 | 1.74 (1.64-1.84) |  |
| Total | 100 days | 2090 | 9826 | 96187 | 108103 | 1.95 (1.87-2.04) |  |
| Alive at 100 days | |  |  |  |  |  |  |
| 1980-2001 | 1 year | 971 | 7924 | 25724 | 34619 | 2.84 (2.67-3.02) | 0.0013 |
| 2002-2015 | 1 year | 1349 | 12100 | 45160 | 58609 | 2.49 (2.36-2.63) |  |
| Total | 1 year | 2320 | 20024 | 70884 | 93228 | 2.63 (2.52-2.73) |  |
| Alive at 1 year | |  |  |  |  |  |  |
| 1980-2001 | 5 years | 354 | 5218 | 19224 | 24796 | 1.47 (1.33-1.63) | 0.2339 |
| 2002-2015 | 5 years | 502 | 7025 | 29960 | 37487 | 1.61 (1.47-1.75) |  |
| Total | 5 years | 856 | 12243 | 49184 | 62283 | 1.55 (1.45-1.65) |  |

**Tables-S5A. Causes of deaths by main cause, transplant period and calendar period in allo-HSCT patients**

Allogeneic

1. Relapse

| group | timepoint | event | competing | Censored | Patients | Cum Inc (95% CI) | Gray test |
| --- | --- | --- | --- | --- | --- | --- | --- |
| 1980-2001 | 30 days | 81 | 1824 | 29223 | 31128 | 0.26 (0.21-0.32) | 0.0037 |
| 2002-2015 | 30 days | 242 | 2412 | 62007 | 64661 | 0.38 (0.33-0.43) |  |
| Total | 30 days | 323 | 4236 | 91230 | 95789 | 0.34 (0.30-0.38) |  |
| Alive at 30 days | |  |  |  |  |  |  |
| 1980-2001 | 100 days | 616 | 4071 | 24457 | 29144 | 2.12 (1.96-2.29) | <0.0001 |
| 2002-2015 | 100 days | 1783 | 4389 | 54992 | 61164 | 2.96 (2.83-3.10) |  |
| Total | 100 days | 2399 | 8460 | 79449 | 90308 | 2.69 (2.58-2.80) |  |
| Alive at 100 days | |  |  |  |  |  |  |
| 1980-2001 | 1 year | 2362 | 3755 | 18158 | 24275 | 9.85 (9.47-10.23) | <0.0001 |
| 2002-2015 | 1 year | 6392 | 5829 | 40535 | 52756 | 13.18 (12.88-13.48) |  |
| Total | 1 year | 8754 | 9584 | 58693 | 77031 | 12.08 (11.84-12.32) |  |
| Alive at 1 year | |  |  |  |  |  |  |
| 1980-2001 | 5 years | 1831 | 1608 | 14191 | 17630 | 10.71 (10.25-11.18) | <0.0001 |
| 2002-2015 | 5 years | 3714 | 3021 | 26979 | 33714 | 13.26 (12.85-13.67) |  |
| Total | 5 years | 5545 | 4629 | 41170 | 51344 | 12.29 (11.98-12.60) |  |

1. GvHD

| group | timepoint | event | competing | Censored | Patients | Cum Inc (95% CI) | Gray test |
| --- | --- | --- | --- | --- | --- | --- | --- |
| 1980-2001 | 30 days | 204 | 1701 | 29223 | 31128 | 0.66 (0.57-0.75) | <0.0001 |
| 2002-2015 | 30 days | 127 | 2527 | 62007 | 64661 | 0.20 (0.17-0.24) |  |
| Total | 30 days | 331 | 4228 | 91230 | 95789 | 0.35 (0.31-0.39) |  |
| Alive at 30 days | |  |  |  |  |  |  |
| 1980-2001 | 100 days | 1627 | 3060 | 24457 | 29144 | 5.60 (5.34-5.87) | <0.0001 |
| 2002-2015 | 100 days | 1501 | 4671 | 54992 | 61164 | 2.49 (2.37-2.61) |  |
| Total | 100 days | 3128 | 7731 | 79449 | 90308 | 3.50 (3.38-3.62) |  |
| Alive at 100 days | |  |  |  |  |  |  |
| 1980-2001 | 1 year | 1254 | 4863 | 18158 | 24275 | 5.21 (4.94-5.50) | <0.0001 |
| 2002-2015 | 1 year | 2089 | 10132 | 40535 | 52756 | 4.28 (4.10-4.46) |  |
| Total | 1 year | 3343 | 14995 | 58693 | 77031 | 4.58 (4.43-4.74) |  |
| Alive at 1 year | |  |  |  |  |  |  |
| 1980-2001 | 5 years | 504 | 2935 | 14191 | 17630 | 2.95 (2.71-3.22) | <0.0001 |
| 2002-2015 | 5 years | 1055 | 5680 | 26979 | 33714 | 3.85 (3.62-4.08) |  |
| Total | 5 years | 1559 | 8615 | 41170 | 51344 | 3.50 (3.33-3.67) |  |

1. Infection - Total

| group | timepoint | event | competing | Censored | Patients | Cum Inc (95% CI) | Gray test |
| --- | --- | --- | --- | --- | --- | --- | --- |
| 1980-2001 | 30 days | 849 | 1056 | 29223 | 31128 | 2.73 (2.56-2.92) | <0.0001 |
| 2002-2015 | 30 days | 1442 | 1212 | 62007 | 64661 | 2.25 (2.13-2.36) |  |
| Total | 30 days | 2291 | 2268 | 91230 | 95789 | 2.41 (2.31-2.50) |  |
| Alive at 30 days | |  |  |  |  |  |  |
| 1980-2001 | 100 days | 1455 | 3232 | 24457 | 29144 | 5.00 (4.76-5.26) | <0.0001 |
| 2002-2015 | 100 days | 1722 | 4450 | 54992 | 61164 | 2.85 (2.72-2.98) |  |
| Total | 100 days | 3177 | 7682 | 79449 | 90308 | 3.55 (3.43-3.67) |  |
| Alive at 100 days | |  |  |  |  |  |  |
| 1980-2001 | 1 year | 1550 | 4567 | 18158 | 24275 | 6.45 (6.14-6.76) | <0.0001 |
| 2002-2015 | 1 year | 2119 | 10102 | 40535 | 52756 | 4.34 (4.17-4.53) |  |
| Total | 1 year | 3669 | 14669 | 58693 | 77031 | 5.03 (4.88-5.20) |  |
| Alive at 1 year | |  |  |  |  |  |  |
| 1980-2001 | 5 years | 496 | 2943 | 14191 | 17630 | 2.89 (2.65-3.15) | 0.7930 |
| 2002-2015 | 5 years | 808 | 5927 | 26979 | 33714 | 2.85 (2.66-3.05) |  |
| Total | 5 years | 1304 | 8870 | 41170 | 51344 | 2.86 (2.71-3.02) |  |

1. Other causes

| group | timepoint | event | competing | Censored | Patients | Cum Inc (95% CI) | Gray test |
| --- | --- | --- | --- | --- | --- | --- | --- |
| 1980-2001 | 30 days | 703 | 1202 | 29223 | 31128 | 2.26 (2.10-2.43) | <0.0001 |
| 2002-2015 | 30 days | 803 | 1851 | 62007 | 64661 | 1.25 (1.17-1.34) |  |
| Total | 30 days | 1506 | 3053 | 91230 | 95789 | 1.58 (1.50-1.66) |  |
| Alive at 30 days | |  |  |  |  |  |  |
| 1980-2001 | 100 days | 844 | 3843 | 24457 | 29144 | 2.90 (2.71-3.10) | <0.0001 |
| 2002-2015 | 100 days | 1074 | 5098 | 54992 | 61164 | 1.78 (1.67-1.88) |  |
| Total | 100 days | 1918 | 8941 | 79449 | 90308 | 2.14 (2.05-2.24) |  |
|  |  |  |  |  |  |  |  |
| 1980-2001 | 1 year | 747 | 5370 | 18158 | 24275 | 3.11 (2.90-3.33) | 0.0136 |
| 2002-2015 | 1 year | 1358 | 10863 | 40535 | 52756 | 2.79 (2.65-2.94) |  |
| Total | 1 year | 2105 | 16233 | 58693 | 77031 | 2.90 (2.77-3.02) |  |
| Alive at 1 year | |  |  |  |  |  |  |
| 1980-2001 | 5 years | 432 | 3007 | 14191 | 17630 | 2.54 (2.32-2.79) | <0.0001 |
| 2002-2015 | 5 years | 921 | 5814 | 26979 | 33714 | 3.45 (3.23-3.68) |  |
| Total | 5 years | 1353 | 8821 | 41170 | 51344 | 3.09 (2.93-3.26) |  |

1. Cause of death unknown

| group | timepoint | event | competing | Censored | Patients | Cum Inc (95% CI) | Gray test |
| --- | --- | --- | --- | --- | --- | --- | --- |
| 1980-2001 | 30 days | 68 | 1837 | 29223 | 31128 | 0.22 (0.17-0.28) | <0.0001 |
| 2002-2015 | 30 days | 40 | 2614 | 62007 | 64661 | 0.06 (0.05-0.08) |  |
| Total | 30 days | 108 | 4451 | 91230 | 95789 | 0.11 (0.09-0.14) |  |
| Alive at 30 days | |  |  |  |  |  |  |
| 1980-2001 | 100 days | 145 | 4542 | 24457 | 29144 | 0.50 (0.42-0.59) | <0.0001 |
| 2002-2015 | 100 days | 92 | 6080 | 54992 | 61164 | 0.15 (0.12-0.19) |  |
| Total | 100 days | 237 | 10622 | 79449 | 90308 | 0.27 (0.23-0.30) |  |
| Alive at 100 days | |  |  |  |  |  |  |
| 1980-2001 | 1 year | 204 | 5913 | 18158 | 24275 | 0.85 (0.74-0.97) | <0.0001 |
| 2002-2015 | 1 year | 263 | 11958 | 40535 | 52756 | 0.54 (0.48-0.61) |  |
| Total | 1 year | 467 | 17871 | 58693 | 77031 | 0.64 (0.59-0.70) |  |
| Alive at 1 year | |  |  |  |  |  |  |
| 1980-2001 | 5 years | 176 | 3263 | 14191 | 17630 | 1.04 (0.89-1.20) | 0.1443 |
| 2002-2015 | 5 years | 237 | 6498 | 26979 | 33714 | 0.90 (0.79-1.03) |  |
| Total | 5 years | 413 | 9761 | 41170 | 51344 | 0.95 (0.86-1.05) |  |

1. All causes of death

| group | timepoint | event | competing | Censored | Patients | Cum Inc (95% CI) | Gray test |
| --- | --- | --- | --- | --- | --- | --- | --- |
| 1980-2001 | 30 days | 1905 | 0 | 29223 | 3112 | 6.13 (5.87-6.40) | <0.0001 |
| 2002-2015 | 30 days | 2654 | 0 | 62007 | 64661 | 4.14 (3.98-4.29) |  |
| Total | 30 days | 4559 | 0 | 91230 | 95789 | 4.79 (4.65-4.92) |  |
| Alive at 30 days | |  |  |  |  |  |  |
| 1980-2001 | 100 days | 4687 | 0 | 24457 | 29144 | 16.12 (15.70-16.55) | <0.0001 |
| 2002-2015 | 100 days | 6172 | 0 | 54992 | 61164 | 10.23 (9.99-10.47) |  |
| Total | 100 days | 10859 | 0 | 79449 | 90308 | 12.14 (11.93-12.36) |  |
| Alive at 100 days | |  |  |  |  |  |  |
| 1980-2001 | 1 year | 6117 | 0 | 18158 | 24275 | 25.47 (24.92-26.02) | 0.1066 |
| 2002-2015 | 1 year | 12221 | 0 | 40535 | 52756 | 25.14 (24.75-25.52) |  |
| Total | 1 year | 18338 | 0 | 58693 | 77031 | 25.23 (24.92-25.55) |  |
| Alive at 1 year | |  |  |  |  |  |  |
| 1980-2001 | 5 years | 3439 | 0 | 14191 | 17630 | 20.14 (19.54-20.74) | <0.0001 |
| 2002-2015 | 5 years | 6735 | 0 | 26979 | 33714 | 24.31 (23.79-24.83) |  |
| Total | 5 years | 10174 | 0 | 41170 | 51344 | 22.69 (22.30-23.08) |  |

**Tables-S5B. Causes of infectious deaths by etiology, transplant period and calendar period in allo-HSCT patients**

1. Bacterial

| group | timepoint | event | competing | Censored | Patients | Cum Inc (95% CI) | Gray test |
| --- | --- | --- | --- | --- | --- | --- | --- |
| 1980-2001 | 30 days | 160 | 1745 | 29223 | 31128 | 0.52 (0.44-0.60) | <0.0001 |
| 2002-2015 | 30 days | 218 | 2436 | 62007 | 64661 | 0.34 (0.30-0.39) |  |
| Total | 30 days | 378 | 4181 | 91230 | 95789 | 0.40 (0.36-0.44) |  |
| Alive at 30 days | |  |  |  |  |  |  |
| 1980-2001 | 100 days | 135 | 4552 | 24457 | 29144 | 0.46 (0.39-0.55) | <0.0001 |
| 2002-2015 | 100 days | 168 | 6004 | 54992 | 61164 | 0.28 (0.24-0.32) |  |
| Total | 100 days | 303 | 10556 | 79449 | 90308 | 0.34 (0.30-0.38) |  |
| Alive at 100 days | |  |  |  |  |  |  |
| 1980-2001 | 1 year | 199 | 5918 | 18158 | 24275 | 0.83 (0.72-0.95) | <0.0001 |
| 2002-2015 | 1 year | 231 | 11990 | 40535 | 52756 | 0.48 (0.42-0.54) |  |
| Total | 1 year | 430 | 17908 | 58693 | 77031 | 0.59 (0.54-0.65) |  |
| Alive at 1 year | |  |  |  |  |  |  |
| 1980-2001 | 5 years | 122 | 3317 | 14191 | 17630 | 0.71 (0.60-0.85) | 0.0147 |
| 2002-2015 | 5 years | 149 | 6586 | 26979 | 33714 | 0.53 (0.45-0.62) |  |
| Total | 5 years | 271 | 9903 | 41170 | 51344 | 0.60 (0.53-0.67) |  |

1. Viral

| group | timepoint | event | competing | Censored | Patients | Cum Inc (95% CI) | Gray test |
| --- | --- | --- | --- | --- | --- | --- | --- |
| 1980-2001 | 30 days | 37 | 1868 | 29223 | 31128 | 0.12 (0.09-0.16) | 0.0010 |
| 2002-2015 | 30 days | 36 | 2618 | 62007 | 64661 | 0.06 (0.04-0.08) |  |
| Total | 30 days | 73 | 4486 | 91230 | 95789 | 0.08 (0.06-0.10) |  |
| Alive at 30 days | |  |  |  |  |  |  |
| 1980-2001 | 100 days | 217 | 4470 | 24457 | 29144 | 0.75 (0.65-0.85) | <0.0001 |
| 2002-2015 | 100 days | 197 | 5975 | 54992 | 61164 | 0.33 (0.28-0.38) |  |
| Total | 100 days | 414 | 10445 | 79449 | 90308 | 0.46 (0.42-0.51) |  |
| Alive at 100 days | |  |  |  |  |  |  |
| 1980-2001 | 1 year | 197 | 5920 | 18158 | 24275 | 0.82 (0.71-0.94) | <0.0001 |
| 2002-2015 | 1 year | 237 | 11984 | 40535 | 52756 | 0.48 (0.43-0.55) |  |
| Total | 1 year | 434 | 17904 | 58693 | 77031 | 0.59 (0.54-0.65) |  |
| Alive at 1 year | |  |  |  |  |  |  |
| 1980-2001 | 5 years | 36 | 3403 | 14191 | 17630 | 0.21 (0.15-0.29) | 0.6312 |
| 2002-2015 | 5 years | 66 | 6669 | 26979 | 33714 | 0.23 (0.18-0.29) |  |
| Total | 5 years | 102 | 10072 | 41170 | 51344 | 0.22 (0.18-0.27) |  |

1. Fungal

| group | timepoint | event | competing | Censored | Patients | Cum Inc (95% CI) | Gray test |
| --- | --- | --- | --- | --- | --- | --- | --- |
| 1980-2001 | 30 days | 134 | 1771 | 29223 | 31128 | 0.43 (0.36-0.51) | <0.0001 |
| 2002-2015 | 30 days | 77 | 2577 | 62007 | 64661 | 0.12 (0.10-0.15) |  |
| Total | 30 days | 211 | 4348 | 91230 | 95789 | 0.22 (0.19-0.25) |  |
| Alive at 30 days | |  |  |  |  |  |  |
| 1980-2001 | 100 days | 211 | 4476 | 24457 | 29144 | 0.73 (0.63-0.83) | <0.0001 |
| 2002-2015 | 100 days | 104 | 6068 | 54992 | 61164 | 0.17 (0.14-0.21) |  |
| Total | 100 days | 315 | 10544 | 79449 | 90308 | 0.35 (0.32-0.39) |  |
| Alive at 100 days | |  |  |  |  |  |  |
| 1980-2001 | 1 year | 249 | 5868 | 18158 | 24275 | 1.04 (0.91-1.17) | <0.0001 |
| 2002-2015 | 1 year | 189 | 12032 | 40535 | 52756 | 0.39 (0.34-0.45) |  |
| Total | 1 year | 438 | 17900 | 58693 | 77031 | 0.60 (0.55-0.66) |  |
| Alive at 1 year | |  |  |  |  |  |  |
| 1980-2001 | 5 years | 64 | 3375 | 14191 | 17630 | 0.37 (0.29-0.47) | 0.0115 |
| 2002-2015 | 5 years | 70 | 6665 | 26979 | 33714 | 0.24 (0.19-0.30) |  |
| Total | 5 years | 134 | 10040 | 41170 | 51344 | 0.29 (0.24-0.34) |  |

1. Parasitic

| group | timepoint | event | competing | Censored | Patients | Cum Inc (95% CI) | Gray test |
| --- | --- | --- | --- | --- | --- | --- | --- |
| 1980-2001 | 30 days | 4 | 1901 | 29223 | 31128 | 0.01 (0.00-0.03) | 0.1670 |
| 2002-2015 | 30 days | 3 | 2651 | 62007 | 64661 | 0.00 (0.00-0.01) |  |
| Total | 30 days | 7 | 4552 | 91230 | 95789 | 0.01 (0.00-0.02) |  |
| Alive at 30 days | |  |  |  |  |  |  |
| 1980-2001 | 100 days | 26 | 4661 | 24457 | 29144 | 0.09 (0.06-0.13) | <0.0001 |
| 2002-2015 | 100 days | 12 | 6160 | 54992 | 61164 | 0.02 (0.01-0.03) |  |
| Total | 100 days | 38 | 10821 | 79449 | 90308 | 0.04 (0.03-0.06) |  |
| Alive at 100 days | |  |  |  |  |  |  |
| 1980-2001 | 1 year | 38 | 6079 | 18158 | 24275 | 0.16 (0.11-0.22) | <0.0001 |
| 2002-2015 | 1 year | 18 | 12203 | 40535 | 52756 | 0.04 (0.02-0.06) |  |
| Total | 1 year | 56 | 18282 | 58693 | 77031 | 0.08 (0.06-0.10) |  |
| Alive at 1 year | |  |  |  |  |  |  |
| 1980-2001 | 5 years | 3 | 3436 | 14191 | 17630 | 0.02 (0.01-0.05) | 0.5048 |
| 2002-2015 | 5 years | 3 | 6732 | 26979 | 33714 | 0.01 (0.00-0.03) |  |
| Total | 5 years | 6 | 10168 | 41170 | 51344 | 0.01 (0.01-0.03) |  |

1. Mixed

| group | timepoint | event | competing | Censored | Patients | Cum Inc (95% CI) | Gray test |
| --- | --- | --- | --- | --- | --- | --- | --- |
| 1980-2001 | 30 days | 29 | 1876 | 29223 | 31128 | 0.09 (0.06-0.13) | 0.7653 |
| 2002-2015 | 30 days | 64 | 2590 | 62007 | 64661 | 0.10 (0.08-0.13) |  |
| Total | 30 days | 93 | 4466 | 91230 | 95789 | 0.10 (0.08-0.12) |  |
| Alive at 30 days | |  |  |  |  |  |  |
| 1980-2001 | 100 days | 56 | 4631 | 24457 | 29144 | 0.19 (0.15-0.25) | 0.7344 |
| 2002-2015 | 100 days | 110 | 6062 | 54992 | 61164 | 0.18 (0.15-0.22) |  |
| Total | 100 days | 166 | 10693 | 79449 | 90308 | 0.19 (0.16-0.22) |  |
| Alive at 100 days | |  |  |  |  |  |  |
| 1980-2001 | 1 year | 65 | 6052 | 18158 | 24275 | 0.27 (0.21-0.34) | 0.1315 |
| 2002-2015 | 1 year | 165 | 12056 | 40535 | 52756 | 0.34 (0.29-0.39) |  |
| Total | 1 year | 230 | 18108 | 58693 | 77031 | 0.32 (0.28-0.36) |  |
| Alive at 1 year | |  |  |  |  |  |  |
| 1980-2001 | 5 years | 14 | 3425 | 14191 | 17630 | 0.08 (0.05-0.13) | 0.0402 |
| 2002-2015 | 5 years | 45 | 6690 | 26979 | 33714 | 0.15 (0.11-0.20) |  |
| Total | 5 years | 59 | 10115 | 41170 | 51344 | 0.13 (0.10-0.16) |  |

1. Infection type unknown

| group | timepoint | event | competing | Censored | Patients | Cum Inc (95% CI) | Gray test |
| --- | --- | --- | --- | --- | --- | --- | --- |
| 1980-2001 | 30 days | 485 | 1420 | 29223 | 31128 | 1.56 (1.43-1.70) | 0.4314 |
| 2002-2015 | 30 days | 1044 | 1610 | 62007 | 64661 | 1.63 (1.53-1.73) |  |
| Total | 30 days | 1529 | 3030 | 91230 | 95789 | 1.61 (1.53-1.69) |  |
| Alive at 30 days | |  |  |  |  |  |  |
| 1980-2001 | 100 days | 810 | 3877 | 24457 | 29144 | 2.79 (2.60-2.98) | <0.0001 |
| 2002-2015 | 100 days | 1131 | 5041 | 54992 | 61164 | 1.87 (1.77-1.98) |  |
| Total | 100 days | 1941 | 8918 | 79449 | 90308 | 2.17 (2.07-2.27) |  |
| Alive at 100 days | |  |  |  |  |  |  |
| 1980-2001 | 1 year | 802 | 5315 | 18158 | 24275 | 3.34 (3.11-3.57) | <0.0001 |
| 2002-2015 | 1 year | 1279 | 10942 | 40535 | 52756 | 2.62 (2.49-2.77) |  |
| Total | 1 year | 2081 | 16257 | 58693 | 77031 | 2.86 (2.74-2.98) |  |
| Alive at 1 year | |  |  |  |  |  |  |
| 1980-2001 | 5 years | 257 | 3182 | 14191 | 17630 | 1.50 (1.32-1.69) | 0.1279 |
| 2002-2015 | 5 years | 475 | 6260 | 26979 | 33714 | 1.70 (1.55-1.86) |  |
| Total | 5 years | 732 | 9442 | 41170 | 51344 | 1.61 (1.50-1.73) |  |

**Tables-S6A. Causes of deaths by main cause, transplant period and calendar period in auto-HSCT patients**

Autologous

1. Relapse

| group | timepoint | event | competing | Censored | Patients | Cum Inc (95% CI) | Gray test |
| --- | --- | --- | --- | --- | --- | --- | --- |
| 1980-2001 | 30 days | 36 | 351 | 11482 | 11869 | 0.31 (0.22-0.42) | 0.6612 |
| 2002-2015 | 30 days | 23 | 120 | 6690 | 6833 | 0.34 (0.23-0.51) |  |
| Total | 30 days | 59 | 471 | 18172 | 18702 | 0.32 (0.25-0.41) |  |
| Alive at 30 days | |  |  |  |  |  |  |
| 1980-2001 | 100 days | 353 | 473 | 10510 | 11336 | 3.14 (2.83-3.48) | <0.0001 |
| 2002-2015 | 100 days | 133 | 98 | 6228 | 6459 | 2.12 (1.79-2.50) |  |
| Total | 100 days | 486 | 571 | 16738 | 17795 | 2.78 (2.54-3.03) |  |
| Alive at 100 days | |  |  |  |  |  |  |
| 1980-2001 | 1 year | 2073 | 705 | 7566 | 10344 | 20.52 (19.74-21.32) | 0.0018 |
| 2002-2015 | 1 year | 974 | 254 | 4625 | 5853 | 18.31 (17.28-19.37) |  |
| Total | 1 year | 3047 | 959 | 12191 | 16197 | 19.77 (19.15-20.41) |  |
| Alive at 1 year | |  |  |  |  |  |  |
| 1980-2001 | 5 years | 1616 | 517 | 5033 | 7166 | 23.51 (22.51-24.52) | <0.0001 |
| 2002-2015 | 5 years | 589 | 203 | 2981 | 3773 | 18.04 (16.72-19.40) |  |
| Total | 5 years | 2205 | 720 | 8014 | 10939 | 21.77 (20.97-22.59) |  |

1. Infection - Total

| group | timepoint | event | competing | Censored | Patients | Cum Inc (95% CI) | Gray test |
| --- | --- | --- | --- | --- | --- | --- | --- |
| 1980-2001 | 30 days | 198 | 189 | 11482 | 11869 | 1.68 (1.46-1.93) | 0.0014 |
| 2002-2015 | 30 days | 73 | 70 | 6690 | 6833 | 1.09 (0.86-1.36) |  |
| Total | 30 days | 271 | 259 | 18172 | 18702 | 1.47 (1.30-1.65) |  |
| Alive at 30 days | |  |  |  |  |  |  |
| 1980-2001 | 100 days | 231 | 595 | 10510 | 11336 | 2.05 (1.80-2.32) | <0.0001 |
| 2002-2015 | 100 days | 47 | 184 | 6228 | 6459 | 0.74 (0.55-0.98) |  |
| Total | 100 days | 278 | 779 | 16738 | 17795 | 1.58 (1.40-1.77) |  |
| Alive at 100 days | |  |  |  |  |  |  |
| 1980-2001 | 1 year | 359 | 2419 | 7566 | 10344 | 3.54 (3.20-3.92) | <0.0001 |
| 2002-2015 | 1 year | 101 | 1127 | 4625 | 5853 | 1.89 (1.55-2.28) |  |
| Total | 1 year | 460 | 3546 | 12191 | 16197 | 2.97 (2.71-3.25) |  |
| Alive at 1 year | |  |  |  |  |  |  |
| 1980-2001 | 5 years | 199 | 1934 | 5033 | 7166 | 2.90 (2.52-3.31) | <0.0001 |
| 2002-2015 | 5 years | 52 | 740 | 2981 | 3773 | 1.59 (1.20-2.07) |  |
| Total | 5 years | 251 | 2674 | 8014 | 10939 | 2.48 (2.19-2.80) |  |

1. Other causes

| group | timepoint | event | competing | Censored | Patients | Cum Inc (95% CI) | Gray test |
| --- | --- | --- | --- | --- | --- | --- | --- |
| 1980-2001 | 30 days | 140 | 247 | 11482 | 11869 | 1.19 (1.01-1.40) | <0.0001 |
| 2002-2015 | 30 days | 39 | 104 | 6690 | 6833 | 0.58 (0.42-0.79) |  |
| Total | 30 days | 179 | 351 | 18172 | 18702 | 0.97 (0.84-1.12) |  |
| Alive at 30 days | |  |  |  |  |  |  |
| 1980-2001 | 100 days | 209 | 617 | 10510 | 11336 | 1.86 (1.62-2.12) | <0.0001 |
| 2002-2015 | 100 days | 44 | 187 | 6228 | 6459 | 0.70 (0.51-0.93) |  |
| Total | 100 days | 253 | 804 | 16738 | 17795 | 1.44 (1.27-1.62) |  |
| Alive at 100 days | |  |  |  |  |  |  |
| 1980-2001 | 1 year | 267 | 2511 | 7566 | 10344 | 2.63 (2.33-2.96) | 0.0496 |
| 2002-2015 | 1 year | 114 | 1114 | 4625 | 5853 | 2.12 (1.76-2.54) |  |
| Total | 1 year | 381 | 3625 | 12191 | 16197 | 2.46 (2.22-2.71) |  |
| Alive at 1 year | |  |  |  |  |  |  |
| 1980-2001 | 5 years | 217 | 1916 | 5033 | 7166 | 3.18 (2.79-3.62) | 0.2114 |
| 2002-2015 | 5 years | 116 | 676 | 2981 | 3773 | 3.80 (3.16-4.53) |  |
| Total | 5 years | 333 | 2592 | 8014 | 10939 | 3.36 (3.02-3.73) |  |

1. Cause of death unknown

| group | timepoint | event | competing | Censored | Patients | Cum Inc (95% CI) | Gray test |
| --- | --- | --- | --- | --- | --- | --- | --- |
| 1980-2001 | 30 days | 13 | 374 | 11482 | 11869 | 0.11 (0.06-0.19) | 0.8642 |
| 2002-2015 | 30 days | 8 | 135 | 6690 | 6833 | 0.12 (0.06-0.23) |  |
| Total | 30 days | 21 | 509 | 18172 | 18702 | 0.11 (0.07-0.17) |  |
| Alive at 30 days | |  |  |  |  |  |  |
| 1980-2001 | 100 days | 33 | 793 | 10510 | 11336 | 0.29 (0.21-0.41) | 0.0150 |
| 2002-2015 | 100 days | 7 | 224 | 6228 | 6459 | 0.11 (0.05-0.22) |  |
| Total | 100 days | 40 | 1017 | 16738 | 17795 | 0.23 (0.17-0.31) |  |
| Alive at 100 days | |  |  |  |  |  |  |
| 1980-2001 | 1 year | 79 | 2699 | 7566 | 10344 | 0.78 (0.62-0.97) | 0.7379 |
| 2002-2015 | 1 year | 39 | 1189 | 4625 | 5853 | 0.74 (0.53-1.00) |  |
| Total | 1 year | 118 | 3888 | 12191 | 16197 | 0.76 (0.64-0.91) |  |
| Alive at 1 year | |  |  |  |  |  |  |
| 1980-2001 | 5 years | 101 | 2032 | 5033 | 7166 | 1.51 (1.23-1.82) | 0.1635 |
| 2002-2015 | 5 years | 35 | 757 | 2981 | 3773 | 1.14 (0.81-1.57) |  |
| Total | 5 years | 136 | 2789 | 8014 | 10939 | 1.40 (1.18-1.64) |  |

1. All causes of death

| group | timepoint | event | competing | Censored | Patients | Cum Inc (95% CI) | Gray test |
| --- | --- | --- | --- | --- | --- | --- | --- |
| 1980-2001 | 30 days | 387 | 0 | 11482 | 11869 | 3.29 (2.98-3.63) | <0.0001 |
| 2002-2015 | 30 days | 143 | 0 | 6690 | 6833 | 2.14 (1.81-2.50) |  |
| Total | 30 days | 530 | 0 | 18172 | 18702 | 2.87 (2.64-3.12) |  |
| Alive at 30 days | |  |  |  |  |  |  |
| 1980-2001 | 100 days | 826 | 0 | 10510 | 11336 | 7.34 (6.87-7.83) | <0.0001 |
| 2002-2015 | 100 days | 231 | 0 | 6228 | 6459 | 3.67 (3.23-4.16) |  |
| Total | 100 days | 1057 | 0 | 16738 | 17795 | 6.02 (5.68-6.38) |  |
| Alive at 100 days | |  |  |  |  |  |  |
| 1980-2001 | 1 year | 2778 | 0 | 7566 | 10344 | 27.48 (26.61-28.35) | <0.0001 |
| 2002-2015 | 1 year | 1228 | 0 | 4625 | 5853 | 23.06 (21.93-24.21) |  |
| Total | 1 year | 4006 | 0 | 12191 | 16197 | 25.96 (25.27-26.66) |  |
| Alive at 1 year | |  |  |  |  |  |  |
| 1980-2001 | 5 years | 2133 | 0 | 5033 | 7166 | 31.09 (29.99-32.20) | <0.0001 |
| 2002-2015 | 5 years | 792 | 0 | 2981 | 3773 | 24.57 (23.07-26.10) |  |
| Total | 5 years | 2925 | 0 | 8014 | 10939 | 29.01 (28.12-29.91) |  |

**Tables-S6B. Causes of infectious deaths by etiology, transplant period and calendar period in auto-HSCT patients**

1. Bacterial

| group | timepoint | event | competing | Censored | Patients | Cum Inc (95% CI) | Gray test |
| --- | --- | --- | --- | --- | --- | --- | --- |
| 1980-2001 | 30 days | 64 | 323 | 11482 | 11869 | 0.54 (0.42-0.69) | 0.0002 |
| 2002-2015 | 30 days | 12 | 131 | 6690 | 6833 | 0.18 (0.10-0.31) |  |
| Total | 30 days | 76 | 454 | 18172 | 18702 | 0.41 (0.33-0.51) |  |
| Alive at 30 days | |  |  |  |  |  |  |
| 1980-2001 | 100 days | 48 | 778 | 10510 | 11336 | 0.43 (0.32-0.56) | 0.0007 |
| 2002-2015 | 100 days | 8 | 223 | 6228 | 6459 | 0.13 (0.06-0.25) |  |
| Total | 100 days | 56 | 1001 | 16738 | 17795 | 0.32 (0.24-0.41) |  |
| Alive at 100 days | |  |  |  |  |  |  |
| 1980-2001 | 1 year | 108 | 2670 | 7566 | 10344 | 1.07 (0.88-1.28) | <0.0001 |
| 2002-2015 | 1 year | 10 | 1218 | 4625 | 5853 | 0.19 (0.10-0.34) |  |
| Total | 1 year | 118 | 3888 | 12191 | 16197 | 0.76 (0.64-0.91) |  |
| Alive at 1 year | |  |  |  |  |  |  |
| 1980-2001 | 5 years | 59 | 2074 | 5033 | 7166 | 0.86 (0.66-1.10) | 0.0031 |
| 2002-2015 | 5 years | 11 | 781 | 2981 | 3773 | 0.35 (0.18-0.61) |  |
| Total | 5 years | 70 | 2855 | 8014 | 10939 | 0.69 (0.55-0.87) |  |

1. Viral

| group | timepoint | event | competing | Censored | Patients | Cum Inc (95% CI) | Gray test |
| --- | --- | --- | --- | --- | --- | --- | --- |
| 1980-2001 | 30 days | 2 | 385 | 11482 | 11869 | 0.02 (0.00-0.06) | 0.0533 |
| 2002-2015 | 30 days | 5 | 138 | 6690 | 6833 | 0.07 (0.03-0.17) |  |
| Total | 30 days | 7 | 523 | 18172 | 18702 | 0.04 (0.02-0.08) |  |
| Alive at 30 days | |  |  |  |  |  |  |
| 1980-2001 | 100 days | 16 | 810 | 10510 | 11336 | 0.14 (0.09-0.23) | 0.1377 |
| 2002-2015 | 100 days | 4 | 227 | 6228 | 6459 | 0.06 (0.02-0.16) |  |
| Total | 100 days | 20 | 1037 | 16738 | 17795 | 0.11 (0.07-0.17) |  |
| Alive at 100 days | |  |  |  |  |  |  |
| 1980-2001 | 1 year | 17 | 2761 | 7566 | 10344 | 0.17 (0.10-0.26) | 0.0630 |
| 2002-2015 | 1 year | 3 | 1225 | 4625 | 5853 | 0.06 (0.02-0.16) |  |
| Total | 1 year | 20 | 3986 | 12191 | 16197 | 0.13 (0.08-0.20) |  |
| Alive at 1 year | |  |  |  |  |  |  |
| 1980-2001 | 5 years | 10 | 2123 | 5033 | 7166 | 0.15 (0.08-0.26) | 0.1041 |
| 2002-2015 | 5 years | 1 | 791 | 2981 | 3773 | 0.03 (0.00-0.02) |  |
| Total | 5 years | 11 | 2914 | 8014 | 10939 | 0.11 (0.06-0.19) |  |

1. Fungal

| group | timepoint | event | competing | Censored | Patients | Cum Inc (95% CI) | Gray test |
| --- | --- | --- | --- | --- | --- | --- | --- |
| 1980-2001 | 30 days | 25 | 362 | 11482 | 11869 | 0.21 (0.14-0.31) | 0.0504 |
| 2002-2015 | 30 days | 6 | 137 | 6690 | 6833 | 0.09 (0.04-0.19) |  |
| Total | 30 days | 31 | 499 | 18172 | 18702 | 0.17 (0.12-0.24) |  |
| Alive at 30 days | |  |  |  |  |  |  |
| 1980-2001 | 100 days | 26 | 800 | 10510 | 11336 | 0.23 (0.16-0.34) | 0.0097 |
| 2002-2015 | 100 days | 4 | 227 | 6228 | 6459 | 0.06 (0.02-0.16) |  |
| Total | 100 days | 30 | 1027 | 16738 | 17795 | 0.17 (0.12-0.24) |  |
| Alive at 100 days | |  |  |  |  |  |  |
| 1980-2001 | 1 year | 39 | 2739 | 7566 | 10344 | 0.39 (0.28-0.52) | 0.2971 |
| 2002-2015 | 1 year | 15 | 1213 | 4625 | 5853 | 0.28 (0.17-0.46) |  |
| Total | 1 year | 54 | 3952 | 12191 | 16197 | 0.35 (0.27-0.45) |  |
| Alive at 1 year | |  |  |  |  |  |  |
| 1980-2001 | 5 years | 26 | 2107 | 5033 | 7166 | 0.38 (0.25-0.54) | 0.3858 |
| 2002-2015 | 5 years | 9 | 783 | 2981 | 3773 | 0.26 (0.13-0.49) |  |
| Total | 5 years | 35 | 2890 | 8014 | 10939 | 0.34 (0.24-0.47) |  |

1. Parasitic

| group | timepoint | event | competing | Censored | Patients | Cum Inc (95% CI) | Gray test |
| --- | --- | --- | --- | --- | --- | --- | --- |
| 1980-2001 | 30 days | 1 | 386 | 11482 | 11869 | 0.01 (0.00-0.05) | - |
| 2002-2015 | 30 days | 0 | 143 | 6690 | 6833 | 0.00 |  |
| Total | 30 days | 1 | 529 | 18172 | 18702 | 0.01 (0.00-0.03) |  |
| Alive at 30 days | |  |  |  |  |  |  |
| 1980-2001 | 100 days | 4 | 822 | 10510 | 11336 | 0.04 (0.01-0.09) | - |
| 2002-2015 | 100 days | 0 | 231 | 6228 | 6459 | 0.00 |  |
| Total | 100 days | 4 | 1053 | 16738 | 17795 | 0.02 (0.01-0.06) |  |
| Alive at 100 days | |  |  |  |  |  |  |
| 1980-2001 | 1 year | 2 | 2776 | 7566 | 10344 | 0.02 (0.00-0.07) | 0.5346 |
| 2002-2015 | 1 year | 2 | 1226 | 4625 | 5853 | 0.04 (0.01-0.13) |  |
| Total | 1 year | 4 | 4002 | 12191 | 16197 | 0.03 (0.01-0.06) |  |
| Alive at 1 year | |  |  |  |  |  |  |
| 1980-2001 | 5 years | 0 | 2133 | 5033 | 7166 | 0.00 |  |
| 2002-2015 | 5 years | 0 | 792 | 2981 | 3773 | 0.00 |  |
| Total | 5 years | 0 | 2925 | 8014 | 10939 | 0.00 |  |

1. Mixed

| group | timepoint | event | competing | Censored | Patients | Cum Inc (95% CI) | Gray test |
| --- | --- | --- | --- | --- | --- | --- | --- |
| 1980-2001 | 30 days | 11 | 376 | 11482 | 11869 | 0.09 (0.05-0.17) | 0.1176 |
| 2002-2015 | 30 days | 2 | 141 | 6690 | 6833 | 0.03 (0.01-0.11) |  |
| Total | 30 days | 13 | 517 | 18172 | 18702 | 0.07 (0.04-0.12) |  |
| Alive at 30 days | |  |  |  |  |  |  |
| 1980-2001 | 100 days | 16 | 810 | 10510 | 11336 | 0.14 (0.09-0.23) | 0.0666 |
| 2002-2015 | 100 days | 3 | 228 | 6228 | 6459 | 0.05 (0.01-0.14) |  |
| Total | 100 days | 19 | 1038 | 16738 | 17795 | 0.11 (0.07-0.17) |  |
| Alive at 100 days | |  |  |  |  |  |  |
| 1980-2001 | 1 year | 24 | 2754 | 7566 | 10344 | 0.24 (0.16-0.35) | 0.0012 |
| 2002-2015 | 1 year | 1 | 1227 | 4625 | 5853 | 0.02 (0.00-0.11) |  |
| Total | 1 year | 25 | 3981 | 12191 | 16197 | 0.16 (0.11-0.23) |  |
| Alive at 1 year | |  |  |  |  |  |  |
| 1980-2001 | 5 years | 7 | 2126 | 5033 | 7166 | 0.10 (0.05-0.20) | 0.7907 |
| 2002-2015 | 5 years | 4 | 788 | 2981 | 3773 | 0.13 (0.04-0.32) |  |
| Total | 5 years | 11 | 2914 | 8014 | 10939 | 0.11 (0.06-0.19) |  |

1. Infection type unknown

| group | timepoint | event | competing | Censored | Patients | Cum Inc (95% CI) | Gray test |
| --- | --- | --- | --- | --- | --- | --- | --- |
| 1980-2001 | 30 days | 95 | 292 | 11482 | 11869 | 0.81 (0.66-0.98) | 0.5055 |
| 2002-2015 | 30 days | 48 | 95 | 6690 | 6833 | 0.72 (0.54-0.94) |  |
| Total | 30 days | 143 | 387 | 18172 | 18702 | 0.78 (0.66-0.91) |  |
| Alive at 30 days | |  |  |  |  |  |  |
| 1980-2001 | 100 days | 121 | 705 | 10510 | 11336 | 1.07 (0.90-1.28) | <0.0001 |
| 2002-2015 | 100 days | 28 | 203 | 6228 | 6459 | 0.44 (0.30-0.63) |  |
| Total | 100 days | 149 | 908 | 16738 | 17795 | 0.85 (0.72-0.99) |  |
| Alive at 100 days | |  |  |  |  |  |  |
| 1980-2001 | 1 year | 169 | 2609 | 7566 | 10344 | 1.67 (1.43-1.93) | 0.0835 |
| 2002-2015 | 1 year | 70 | 1158 | 4625 | 5853 | 1.31 (1.03-1.64) |  |
| Total | 1 year | 239 | 3767 | 12191 | 16197 | 1.55 (1.36-1.75) |  |
| Alive at 1 year | |  |  |  |  |  |  |
| 1980-2001 | 5 years | 97 | 2036 | 5033 | 7166 | 1.42 (1.16-1.72) | 0.0139 |
| 2002-2015 | 5 years | 27 | 765 | 2981 | 3773 | 0.83 (0.56-1.19) |  |
| Total | 5 years | 124 | 2801 | 8014 | 10939 | 1.23 (1.03-1.46) |  |

**Tables-7A. Cumulative incidences of deaths depending on cause, cohort, period after transplant, and calendar period in allo-HSCT patients**

|  |  |  |  |  |  | Cum Inc (95% CI) | | | |  |
| --- | --- | --- | --- | --- | --- | --- | --- | --- | --- | --- |
| relapse | group | event | competing | Censored | Patients | 30 days | 100 days | 1 year | 5 years | Gray test |
|  | 1980-2001 | 5278 | 12559 | 13291 | 31128 | 0.26 (0.21-0.32) | 2.25 (2.09-2.42) | 10.00 (9.67-10.34) | 16.29 (15.87-16.71) | <0.0001 |
|  | 2002-2015 | 12371 | 16215 | 36075 | 64661 | 0.38 (0.33-0.43) | 3.22 (3.08-3.36) | 14.56 (14.27-14.85) | 23.10 (22.73-23.47) |  |
|  | Total | 17649 | 28774 | 49366 | 95789 | 0.34 (0.30-0.38) | 2.90 (2.79-3.01) | 13.00 (12.78-13.23) | 20.69 (20.41-20.97) |  |
|  |  |  |  |  |  |  |  |  |  |  |
| gvhd | group | event | competing | Censored | Patients | 30 days | 100 days | 1 year | 5 years | Gray test |
|  | 1980-2001 | 3763 | 14074 | 13291 | 31128 | 0.66 (0.57-0.75) | 5.91 (5.65-6.18) | 10.02 (9.68-10.35) | 11.75 (11.39-12.11) | <0.0001 |
|  | 2002-2015 | 4898 | 23688 | 36075 | 64661 | 0.20 (0.17-0.24) | 2.58 (2.46-2.71) | 6.27 (6.07-6.46) | 8.74 (8.50-8.99) |  |
|  | Total | 8661 | 37762 | 49366 | 95789 | 0.35 (0.31-0.39) | 3.68 (3.56-3.80) | 7.51 (7.34-7.68) | 9.70 (9.50-9.90) |  |
|  |  |  |  |  |  |  |  |  |  |  |
| infection | group | event | competing | Censored | Patients | 30 days | 100 days | 1 year | 5 years | Gray test |
|  | 1980-2001 | 4475 | 13362 | 13291 | 31128 | 2.73 (2.56-2.92) | 7.43 (7.14-7.73) | 12.51 (12.14-12.88) | 14.20 (13.82-14.60) | <0.0001 |
|  | 2002-2015 | 6178 | 22408 | 36075 | 64661 | 2.25 (2.13-2.36) | 4.98 (4.81-5.15) | 8.72 (8.49-8.94) | 10.56 (10.30-10.81) |  |
|  | Total | 10653 | 35770 | 49366 | 95789 | 2.41 (2.31-2.50) | 5.79 (5.64-5.94) | 10.00 (9.80-10.19) | 11.79 (11.58-12.00) |  |
|  |  |  |  |  |  |  |  |  |  |  |
| other causes | group | event | competing | Censored | Patients | 30 days | 100 days | 1 year | 5 years | Gray test |
|  | 1980-2001 | 3380 | 14457 | 13291 | 31128 | 2.26 (2.10-2.43) | 4.99 (4.75-5.23) | 7.44 (7.15-7.73) | 8.93 (8.61-9.25) | <0.0001 |
|  | 2002-2015 | 4416 | 24170 | 36075 | 64661 | 1.25 (1.17-1.34) | 2.95 (2.82-3.09) | 5.36 (5.18-5.54) | 7.58 (7.35-7.81) |  |
|  | Total | 7796 | 38627 | 49366 | 95789 | 1.58 (1.50-1.66) | 3.62 (3.50-3.74) | 6.04 (5.89-6.20) | 7.97 (7.79-8.16) |  |
|  |  |  |  |  |  |  |  |  |  |  |
| unknown | group | event | competing | Censored | Patients | 30 days | 100 days | 1 year | 5 years | Gray test |
|  | 1980-2001 | 941 | 16896 | 13291 | 31128 | 0.22 (0.17-0.28) | 0.69 (0.60-0.78) | 1.36 (1.23-1.49) | 1.96 (1.81-2.13) | <0.0001 |
|  | 2002-2015 | 723 | 27863 | 36075 | 64661 | 0.06 (0.05-0.08) | 0.21 (0.18-0.25) | 0.68 (0.61-0.74) | 1.26 (1.16-1.36) |  |
|  | Total | 1664 | 44759 | 49366 | 95789 | 0.11 (0.09-0.14) | 0.37 (0.33-0.41) | 0.90 (0.84-0.97) | 1.50 (1.42-1.59) |  |
|  |  |  |  |  |  |  |  |  |  |  |
| all deaths | group | event | competing | Censored | Patients | 30 days | 100 days | 1 year | 5 years | Gray test |
|  | 1980-2001 | 17837 | 0 | 13291 | 31128 | 6.13 (5.87-6.40) | 21.27 (20.81-21.72) | 41.32 (40.77-41.87) | 53.13 (52.57-53.70) | <0.0001 |
|  | 2002-2015 | 28586 | 0 | 36075 | 64661 | 4.14 (3.98-4.29) | 13.94 (13.67-14.21) | 35.57 (35.18-35.96) | 51.23 (50.79-51.68) |  |
|  | Total | 46423 | 0 | 49366 | 95789 | 4.79 (4.65-4.92) | 16.35 (16.11-16.59) | 37.46 (37.14-37.78) | 51.65 (51.30-51.99) |  |

**Tables-7B. Cumulative incidences of deaths depending on cause, cohort, period after transplant, and calendar period in auto-HSCT patients**

|  |  | |  |  |  | Cum Inc (95% CI) | | | |  |
| --- | --- | --- | --- | --- | --- | --- | --- | --- | --- | --- |
| relapse | group | event | competing | Censored | Patients | 30 days | 100 days | 1 year | 5 years | Gray test |
|  | 1980-2001 | 4349 | 2384 | 5136 | 11869 | 0.31 (0.22-0.42) | 3.34 (3.03-3.68) | 21.73 (20.98-22.50) | 37.01 (36.10-37.92) | <0.0001 |
|  | 2002-2015 | 1778 | 734 | 4321 | 6833 | 0.34 (0.23-0.51) | 2.42 (2.07-2.82) | 19.69 (18.66-20.73) | 32.77 (31.47-34.08) |  |
|  | Total | 6127 | 3118 | 9457 | 18702 | 0.32 (0.25-0.41) | 3.02 (2.77-3.27) | 21.06 (20.45-21.68) | 35.78 (35.03-36.52) |  |
|  |  |  |  |  |  |  |  |  |  |  |
| infection | group | event | competing | Censored | Patients | 30 days | 100 days | 1 year | 5 years | Gray test |
|  | 1980-2001 | 1011 | 5722 | 5136 | 11869 | 1.68 (1.46-1.93) | 3.67 (3.34-4.02) | 6.84 (6.39-7.31) | 8.72 (8.21-9.26) | <0.0001 |
|  | 2002-2015 | 277 | 2235 | 4321 | 6833 | 1.09 (0.86-1.36) | 1.82 (1.51-2.16) | 3.60 (3.15-4.08) | 4.75 (4.21-5.33) |  |
|  | Total | 1288 | 7957 | 9457 | 18702 | 1.47 (1.30-1.65) | 3.00 (2.76-3.26) | 5.72 (5.38-6.07) | 7.39 (7.00-7.79) |  |
|  |  |  |  |  |  |  |  |  |  |  |
| other causes | group | event | competing | Censored | Patients | 30 days | 100 days | 1 year | 5 years | Gray test |
|  | 1980-2001 | 1018 | 5715 | 5136 | 11869 | 1.19 (1.01-1.40) | 2.99 (2.69-3.31) | 5.34 (4.94-5.77) | 7.41 (6.94-7.91) | 0,001 |
|  | 2002-2015 | 356 | 2156 | 4321 | 6833 | 0.58 (0.42-0.79) | 1.26 (1.02-1.56) | 3.27 (2.84-3.74) | 6.02 (5.38-6.71) |  |
|  | Total | 1374 | 7871 | 9457 | 18702 | 0.97 (0.84-1.12) | 2.37 (2.15-2.60) | 4.61 (4.31-4.93) | 6.88 (6.50-7.28) |  |
|  |  |  |  |  |  |  |  |  |  |  |
| unknown | group | event | competing | Censored | Patients | 30 days | 100 days | 1 year | 5 years | Gray test |
|  | 1980-2001 | 355 | 6378 | 5136 | 11869 | 0.11 (0.06-0.19) | 0.39 (0.29-0.52) | 1.09 (0.92-1.30) | 2.07 (1.82-2.35) | 0,0706 |
|  | 2002-2015 | 101 | 2411 | 4321 | 6833 | 0.12 (0.06-0.23) | 0.23 (0.13-0.37) | 0.92 (0.70-1.19) | 1.75 (1.41-2.15) |  |
|  | Total | 456 | 8789 | 9457 | 18702 | 0.11 (0.07-0.17) | 0.34 (0.26-0.43) | 1.03 (0.89-1.19) | 1.98 (1.77-2.20) |  |
|  |  |  |  |  |  |  |  |  |  |  |
| all deaths | group | event | competing | Censored | Patients | 30 days | 100 days | 1 year | 5 years | Gray test |
|  | 1980-2001 | 6733 | 0 | 5136 | 11869 | 3.29 (2.98-3.63) | 10.39 (9.84-10.95) | 35.02 (34.14-35.89) | 55.22 (54.28-56.15) | <0.0001 |
|  | 2002-2015 | 2512 | 0 | 4321 | 6833 | 2.14 (1.81-2.50) | 5.73 (5.18-6.31) | 27.47 (26.32-28.63) | 45.29 (43.88-46.69) |  |
|  | Total | 9245 | 0 | 9457 | 18702 | 2.87 (2.64-3.12) | 8.72 (8.32-9.14) | 32.42 (31.72-33.13) | 52.03 (51.24-52.81) |  |
